# Sustainable UV-Crosslinkable Acrylic Pressure-Sensitive Adhesives for Medical Application

**DOI:** 10.3390/ijms222111840

**Published:** 2021-10-31

**Authors:** Paula Ossowicz-Rupniewska, Paulina Bednarczyk, Małgorzata Nowak, Anna Nowak, Wiktoria Duchnik, Łukasz Kucharski, Joanna Rokicka, Adam Klimowicz, Zbigniew Czech

**Affiliations:** 1 Department of Chemical Organic Technology and Polymeric Materials, Faculty of Chemical Technology and Engineering, West Pomeranian University of Technology in Szczecin, Piastów Ave. 42, 71-065 Szczecin, Poland; bednarczyk.pb@gmail.com (P.B.); gosia.nowak.zut@gmail.com (M.N.); joanna.rokicka@zut.edu.pl (J.R.); psa_czech@wp.pl (Z.C.); 2 Department of Cosmetic and Pharmaceutical Chemistry, Pomeranian Medical University in Szczecin, Powstańców Wielkopolskich Ave. 72, 70-111 Szczecin, Poland; anowak@pum.edu.pl (A.N.); wiktoria.duchnik@pum.edu.pl (W.D.); lukasz.kucharski@pum.edu.pl (Ł.K.); adklim@pum.edu.pl (A.K.)

**Keywords:** acrylic pressure-sensitive adhesives, transdermal patch, isobornyl methacrylate, shear strength, adhesion, tack

## Abstract

This study aimed to investigate the potential of photoreactive acrylate patches as systems for transdermal drug delivery, in particular, using more renewable alternatives and more environmentally friendly synthesis routes of transdermal patches. Therefore, the aim of this study was to develop a transdermal patch containing ibuprofen and investigate its performance in vitro through the pigskin. Transparent patches were prepared using four acrylate copolymers with an incorporated photoinitiator. Two types of transdermal patches based on the photocrosslinking acrylic prepolymers with isobornyl methacrylate as biocomponent and monomer increasing Tg (“hard”) were manufactured. The obtained patches were characterized for their adhesive properties and tested for permeability of the active substance. It turns out that patches whose adhesive matrix is photoreactive polyacrylate copolymers have a higher cohesion than patches from commercial adhesives, while the modification of the copolymers with isobornyl methacrylate resulted in an improvement in adhesion and tack. This study demonstrates the feasibility of developing photoreactive acrylic-based transdermal patches that contain biocomponents that can deliver a therapeutically relevant dose of ibuprofen.

## 1. Introduction

Transdermal drug delivery is one of the most important methods of delivering the drug to the body [1]. This is important especially in the use of NSAIDs, including ibuprofen (IBU), because their oral absorption could be associated with severe gastric irritation leading to gastric bleeding and ulcers. Therefore, transdermal delivery is preferred as it bypasses hepatic first-pass metabolism [1,2]. Moreover, advantages associated with transdermal delivery include also non-invasive delivery, reduced dosing frequency as well as constant levels of the drug in the plasma [3]. In recent years, when designing various types of transdermal systems, including patches, more and more attention has been paid to the use of natural materials and more environmentally friendly.

There are generally two types of transdermal patches, namely, matrix (drug-in-adhesive) and reservoir. The matrix-based transdermal patch, which was the system of choice in this study, consists of a release liner, an adhesive matrix, and an impermeable backing membrane. One of the significant advantages of matrix type transdermal patches, when compared to reservoir patches, is the ease of use and manufacturability, as well as the acceptable cost of the products, and absence of dose dumping [4]. The matrix transdermal patches are usually prepared using organic solvent-based pressure-sensitive adhesives (PSAs), such as acrylate copolymer, silicone, polyisobutylene (PIB). However, acrylate copolymers have an advantage over the others due to their good wettability to the substrate and good solubility of active substances. 

PSAs are soft polymeric materials that show permanent stickiness at room temperature and instantly adhere to surfaces when mild pressure is applied. The adhesion performances can be regulated by the copolymer formulation, which will determine the glass transition temperature (Tg) and the shear modulus (G) [5]. The acrylic PSAs offer the highest balance of adhesion, cohesion, and excellent water resistance [6,7,8]. Typically, an acrylic PSA consists of 70 to 90% of a “soft” acrylate (e.g., 2-ethylhexyl acrylate) [9]. A high amount of such monomer is needed to bring the Tg of the material down to values ensuring a good tack, typically between −5 °C and −60 °C. To ensure elasticity and strength of the joint, 10 to 30% of a “hard” acrylate (e.g., methyl methacrylate) is built in to increase the final Tg [5,10,11]. The performance requirements of medical PSAs based on acrylics are demanding as their ability to adhere well to varying skin types (both dry and moist), they must be removable without leaving adhesive residue or causing skin damage, and should not irritate the skin. Ideally, medical PSA adheres strongly to the skin but can be easily removed with little or no trauma (adhesion properties) and without adhesive residues (cohesion properties) [6]. UV-crosslinkable acrylic PSA is becoming increasingly important due to the environmental hazards and medical applications associated with conventional crosslinkable solvent-borne PSAs and the performance shortcomings of PSAs based on aqueous systems [6].

The commercial PSAs are mostly based on petroleum resources [12]. This dependence is an increasing concern in today’s environmentally conscious society. Due to the anticipated limitations in the crude oil reserves and the design of policy-influencing documents in favor of a biobased and sustainable economy, the industry is forced to seek greater sustainability both in resource and in processes [5,13] without compromising the properties of the end-products [14,15,16]. In the past years, several reports on new and more renewable building blocks for PSAs have been published, in particular renewable raw materials that could feasibly be developed for replacing traditional petroleum-based monomers in PSA formulation [12]. Imam et al. [17] and Vendamme [18] have provided an overview of recent developments in the field of PSAs derived from renewable building blocks. Wool et al. [19,20] developed bio-based PSAs from acrylated methyl oleate (AMO, from soybean oil) using emulsion and miniemulsion polymerization techniques. On the other hand, Sun et al. [21] explored a concept for novel bio-based PSAs derived from soybean oil intending to improve thermal stability, transparency, and peel strength for use in optoelectronic applications. Next to these resources, terpenes prove to be a wide and diverse class of renewable organic compounds that include a large structural and functional variety [5,22]. The use of terpenes in acrylic PSA applications has already been reported. Baek et al. used tetrahydrogeranyl acrylate (THGA) combined with isobornyl [23], or menthyl [24] acrylate, to obtain optically clear pressure-sensitive adhesives via UV light-triggered photocopolymerisation in bulk. The use of isobornyl methacrylate was reported in a study by Zhang et al. [25]. Moreover, IBOMA, as well as isobornyl acrylate (IBOA), was also used by Badía and coworkers to synthesize PSAs [10]. Terpene-based chemicals have the potential of becoming more important as an alternative to both soft and hard monomers [26,27,28]. In the literature review, there are no reports on the use of isobornyl methacrylate as a bio-raw material for use in photo-crosslinked adhesives for medical patches.

This study aimed to develop and characterize transdermal patches for the delivery of ibuprofen and investigate its permeation through the human epidermis. The adhesive layer consisted of photoreactive polyacrylate copolymers with renewable sources (IBOMA). The major disadvantage of suitable acrylic PSAs in skin-contact applications is the high peel force required to remove them, reflecting their low cohesive strength. To reduce this problem, in addition to the high Tg biomonomer [29], into polymer chain special crosslinking agent in form of an unsaturated photoinitiator was incorporated. Additionally, this study aimed to investigate the potential of photoreactive acrylate patches as systems for transdermal drug delivery for ibuprofen, in particular, using more renewable alternatives and more environmentally friendly synthesis routes of transdermal patches. In this work, (meth)acrylate derivatives of terpenoids, i.e., isoborneol are introduced in the synthesis of solvent-borne pressure-sensitive adhesives (PSA) to obtain transdermal patches. The biobased PSAs are found to be comparable in terms of tack, peel strength, and shear resistance to benchmark petroleum-derived PSAs. The increasing regulations and restrictions in favor of a biobased and sustainable community could compete with the strong economic position of the polymer industry, which is still heavily relying on crude oil. The objective was also extended to evaluate the transdermal matrix patch based on the in vitro drug permeation profile across dermatomed pigskin, and the adhesion properties of the patch. The objective of the study was also extended to evaluate the transdermal matrix patch based on the in vitro drug permeation profile across pigskin as well as the adhesion properties of the patch.

## 2. Results and Discussion

### 2.1. Evaluation of Tack, Peel Adhesion, and Shear Strength of Photocrosslinked PSA

It is known from the literature that the cross-linking process is one of the most important factors in modifying the properties of pressure-sensitive adhesives, especially in order to obtain a material with a well-defined profile. Therefore, the next part of the research concerns the determination of the influence of the cross-linking process on the adhesive films properties of the commercial and newly formed acrylate copolymers (Table 1). For this purpose, the obtained adhesive films were cross-linked under varying conditions of UV radiation time while maintaining a constant setting of the adhesive film thickness (250 µm). Model polyacrylate adhesives were used for the study, i.e., DURO-TAK 387-2516 (DT16) or DURO-TAK 378-2054 (DT54), and compared with the newly obtained acrylate copolymers. The adhesive layers obtained from commercial adhesives were thermally cross-linked at 110 °C and for 10 min, while the adhesive layers based on the obtained adhesives were first dried at 110 °C and for 10 min, and then cross-linked under a UV lamp using a different dose of radiation.

The shear strength of the obtained adhesive layers was high with the exception of the adhesive layer obtained from a copolymer containing hydroxyl groups, i.e., a copolymer containing 2-hydroxyethyl acrylate (BK-H and BK-H-I) in its structure. However, increasing the cross-linking time by means of UV radiation improved the cohesion. Moreover, the modification of the adhesive with the use of a biomonomer (BK-H-I) also resulted in obtaining higher cohesion, which also improved with increasing radiation dose during cross-linking. Ideally, medical PSA adheres strongly to the skin but can be easily removed without adhesive residues (cohesion properties) and with little or no trauma (adhesion properties). Thus, highly critical PSA adhesive properties are shear strength and peel adhesion. Despite the positive effect of the cross-linking dose on the adhesive cohesion, a reduction in the adhesion and tackiness of the adhesive layers was also observed. This is probably due to an increase in the cross-link density of the adhesive film. A positive effect of the presence of the biomonomer on adhesion and tack was also observed. Among other several polyacrylates, generally with a low glass transition temperature (*T*_g_), poly(isobornyl acrylate) (PIBA) has several interesting physical properties such as high glass transition temperature (*T*_g_) (94 °C) and hardness (19.6 kg/mm^2^ at 20°). The bulky side group of isobornyl acrylate is responsible for the high *T*_g_, comparable with those of poly(methyl methacrylate) (PMMA, *T*_g_ = 105 °C) and poly(styrene) (PS, *T*_g_ = 100 °C) [29]. Thus, the modification of the adhesive with isobornyl acrylate increased the glass transition temperature of the copolymer and improved the self-adhesive properties. Furthermore, adhesiveness is a fundamental property of transdermal patches that is essentially required to ensure complete contact between the entire surface area of the patch and skin during the wear period, for the efficient delivery of drugs [4,30]. The highest adhesion and tack showed the adhesive layer obtained from a copolymer with hydroxyl groups and modified with isobornyl acrylate (BK-H-I). The values of these parameters were higher than commercial adhesives. The obtained UV-crosslinkable solvent-borne acrylic systems contain photoreactive groups as an unsaturated photoinitiator incorporated into the polymer chain during PSA synthesis. Photoreactive solvent-borne acrylic PSA with biocomponents are not commercially available on the market for the production of pressure-sensitive adhesive tapes, labels, and medical products. Thus, the presented layers of adhesive films are sufficient to obtain medical plasters with the required criteria, therefore they were tested with the drug in the next step.

### 2.2. Development of the Transdermal Patch

**Figure 1:**
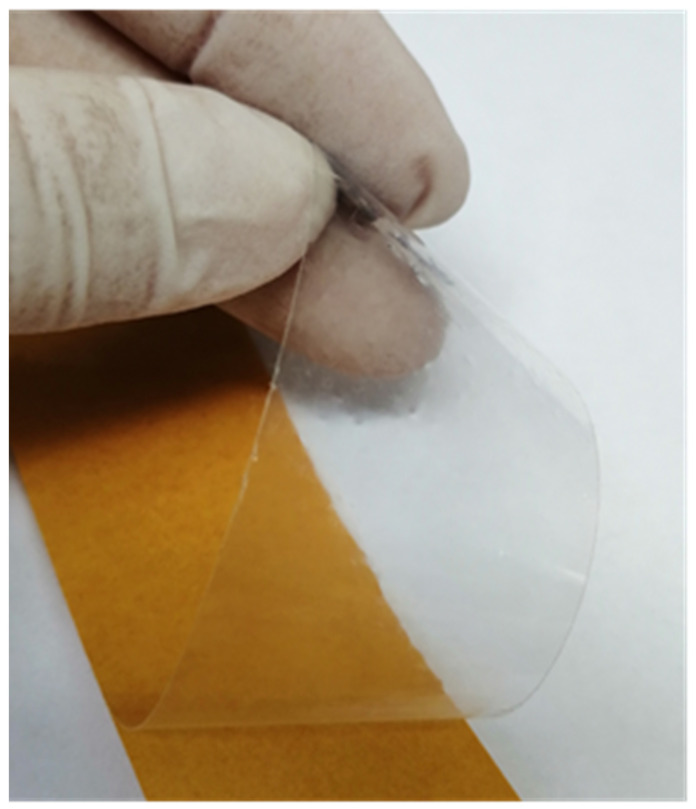
Example photo of the obtained transdermal patch.

Transdermal drug delivery systems (TDDS) are employed for the delivery of drugs across the skin, into the systemic circulation. Adhesion of a TDDS to the skin is a critical factor that affects its performance. The entire delivery surface of a TDDS must be in complete contact with the skin, as the partitioning of the drug between the TDDS and skin is the driving force for permeation [31]. Apart from adhesion, the PSA also affects other critical quality attributes of the TDDS such as tack, shear strength, as well as drug delivery, and flux across the skin, making it critical to the efficacy and quality of the finished product. However, the adhesives industry is highly dependent on finite fossil resources and complicated technological processes. The industry is forced to seek greater sustainability both in resource and in processes without compromising the properties of the end-products [5]. The selection of a suitable PSA is thus pivotal in the development of a transdermal patch. A good adhesive is one that does not leave any residue upon removal, is easy to use, stable to environmental changes, non-irritant, and non-sensitive to the skin, compatible with other formulation components, allows sufficient drug solubility, and possesses the necessary adhesive properties, such as tack, shear, and skin adhesion [4,32]. In our study, we employed various adhesive matrices based on commercial or synthetized acrylate copolymers without and with the use of biocomponent (IBOMA) as well as with the use of various functional monomers (AA or 2-EHA). Drug substance (IBU) has been dispersed in the adhesive matrix, which resulted in transparent patches (Figure 1).

Shear strength (shear adhesion) reveals the resistance of a transdermal patch to tangential stresses and, therefore, the cohesion of the matrix [33]. In this study, the parallel force required to pull a fixed area of the patch (2.5 cm × 2.5 cm) from a standard flat surface (stainless steel) was tested. The average time taken for the patch to drop from the test surface was found to be from 3 to 10 min for commercial adhesives, and from 4 to 15 min for an obtained adhesive matrix with AA as a functional monomer and above 1 h for an obtained adhesive matrix with 2-HEA as a functional monomer (Table 2). The higher cohesion of the AA series copolymers may be due to the higher T_g_ of the starting homomonomer as compared to the 2-HEA copolymer). The presence of IBOMA structure in copolymers results in a higher network density, which is reflected in the achievement of higher cohesion compared to other synthesized and commercial acrylate copolymers. The higher cohesion of the obtained photoreactive transdermal patches compared to patches based on commercial thermally cross-linked acrylate copolymers may be related to the possibility of obtaining dense polymer networks due to the presence of an unsaturated photoinitiator and the use of UV radiation energy.

An ideal transdermal patch should peel off without causing delamination, but also should have good adhesion to the skin. Among all tested transdermal patches, the highest adhesion was characterized by the patch, the adhesive matrix of which was the BK-A-I adhesive, i.e., based on a copolymer with acrylic acid and isobornyl methacrylate. In turn, patches whose adhesive matrix did not have isobornyl methacrylate had the lowest adhesion (BK-A-IBU and BK-H-IBU).

The adhesion efficiency of a transdermal patch can be tested by tack evaluation methods, which measure the force of debonding on the application of light pressure, for a short duration of time. The highest tray was characterized by patches whose adhesive matrix was commercial adhesives. As in the case of adhesion, patches of unmodified IBOMA adhesives had the lowest tack.

The thermal stability of the adhesive layer of the patches was investigated without and with the active substance. The onset decomposition temperature and temperature corresponding to the weight loss of 50% (determined from TG curves) and maximum decomposition temperatures (determined from DTG curves) were determined and presented in Table 3 and Supplementary Figures S1–S12 (Supplementary Materials). It was found that all ibuprofen-containing adhesives have lower decomposition temperatures than those without the active ingredient, which is related to the thermal stability of this additive. Commercial adhesives and BK-H-I are stable up to a temperature of about 300 °C, while the other adhesives obtained in this research have lower decomposition temperatures by about 80 °C. In a group of various adhesives without ibuprofen, the values of onset decomposition temperatures were in the range of 219.7 °C for BK-H up to 326.6 °C for BK-A. The adhesives with ibuprofen have onset decomposition temperature in the range 153.7–170.2 °C.

The values of temperatures corresponding to a 50% weight loss of adhesives without ibuprofen were from 340.5 to 380.8 °C, while for ibuprofen adhesives—form 322.2 to 363.1 °C. 

For the majority of adhesives, the maximum decomposition temperatures were highest than temperatures corresponding to 50% weight loss. For various adhesives tested, it was shown that the maximum decomposition temperature falls within a range of 329.4–393.5 °C.

### 2.3. Evaluation of In Vitro Drug Permeation Studies

**Figure 2:**
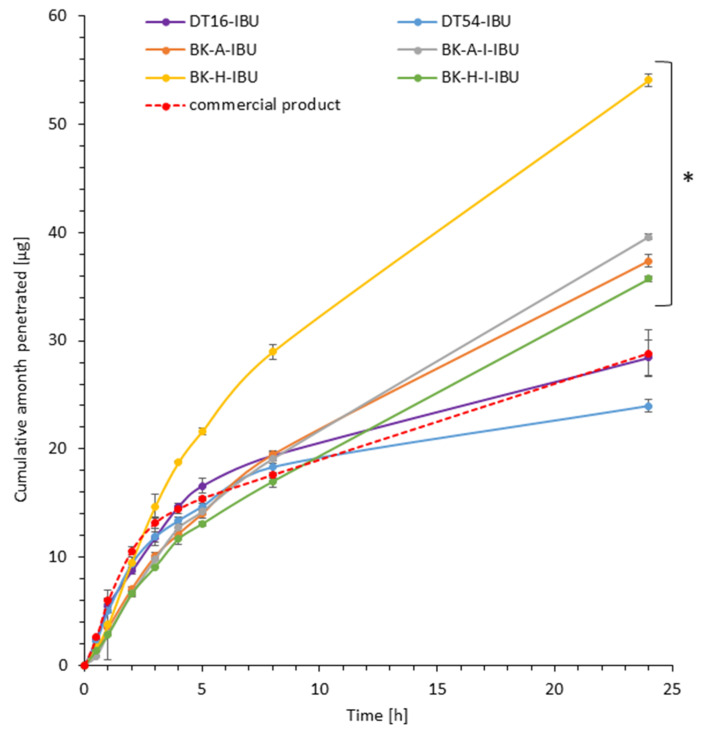
Time course of the cumulative amount of ibuprofen through pigskin. Each point represents the mean ± SD (*n* = 3), α = 0.05. For * *p* < 0.0001 versus the control (commercial product). The statistically significant difference was estimated using the ANOVA test (Tukey’s test).

**Figure 3:**
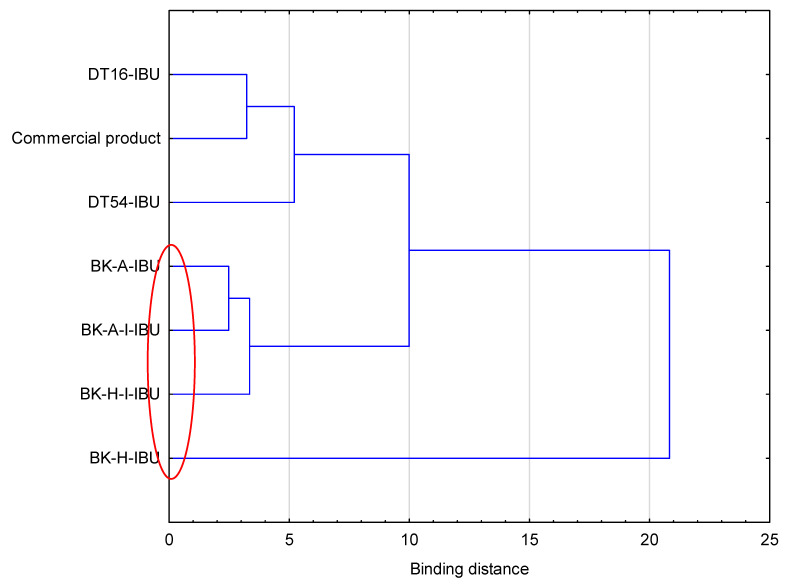
Cluster analysis graph for the mean accumulated mass of ibuprofen during 24 h penetration. The patches with similar penetration are marked with a red circle.

In our in vitro study, the penetration of ibuprofen from different obtained patches with acrylic PSAs was compared with the commercial product. Results of the in vitro efficiency permeation experiments related to ibuprofen are summarized in Table 4. The permeation profiles of ibuprofen from different patches through pigskin are shown in Figure 2 and Figure 3.

It was shown that the patches with the obtained acrylic PSA are an excellent alternative to those with commercial adhesives and even to the commercial ibuprofen patch. All permeation parameters are similar or even better for z patches with obtained acrylic PSA. The patches have been shown to allow the penetration of more of the active substance for all patches obtained with adhesives based on 2-ethylhexyl acrylate. These values have been shown to be from 1.24 for BK-H-I-IBU to 1.88 for BK-H-IBU times higher than for the commercial product.

The highest ibuprofen transdermal fluxes were attained for BK-H-IBU (5.338 µg/cm^2^∙h) and the lowest with the DT54-IBU (4.702 µg/cm^2^∙h) patches. Permeability coefficients (K_P_) were in the range from 2.478∙10^3^ for BK-H-I-IBU to 3.737∙10^3^ cm/h for BK-H-IBU. The ability of the drug to escape from the vehicle and move into the outermost layers of the stratum corneum (K_m_) ranged from 1.276 to 5.261 for commercial product and BK-H-I-IBU, respectively.

**Figure 4:**
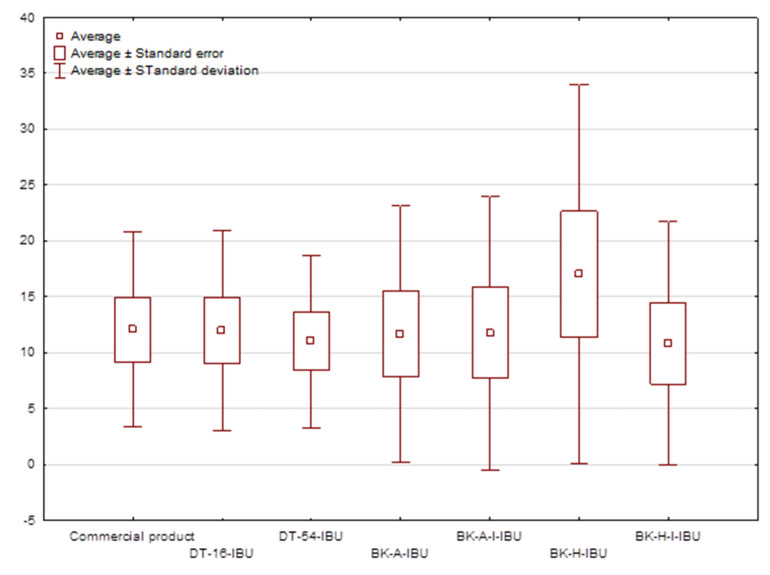
The box plot of cumulative mass for IBU during 24 h penetration.

The cumulative mass in acceptor fluid, considering all time points, is presented in Figure 2, which clearly shows the higher release of the active substance from the patch obtained from 2-ethylhexyl acrylate, ethyl acrylate, and 2-hydroxyethyl acrylate (BK-H-IBU). The cumulative mass of IBU, determined after 24 h of permeation, was as follows: BK-H-IBU > BK-A-I-IBU > BK-A-IBU > BK-H-I-IBU > commercial product > DT16-IBU > and DT54-IBU. In the case of adhesives containing 2-ethylhexyl acrylate, ethyl acrylate, and 2-hydroxyethyl acrylate, significantly higher penetrations of ibuprofen were demonstrated compared to commercial adhesives as well as control (commercial product). This is also confirmed by the cluster analysis test, in which these compounds form a separate cluster (red circle). They are characterized by a similar penetration when analyzing the entire 24-h study—Figure 3. Whereas, the box-plot shows the course of the average cumulative mass collected from all time points, where the most favorable of drug penetration with BK-H-IBU is clearly visible—Figure 4.

For drugs NSAIDs, among others, ibuprofen’s faster permeation is preferable to achieve a rapid therapeutic effect, which causes a faster decrease in inflammation in the underlying tissues [34]. However, due to the oft use of this group of drugs in the relief of chronic pain, also gradual penetration over a longer period is desirable. Moreover, there is also a large group of other drugs requiring sustained release kinetics. 

**Figure 5:**
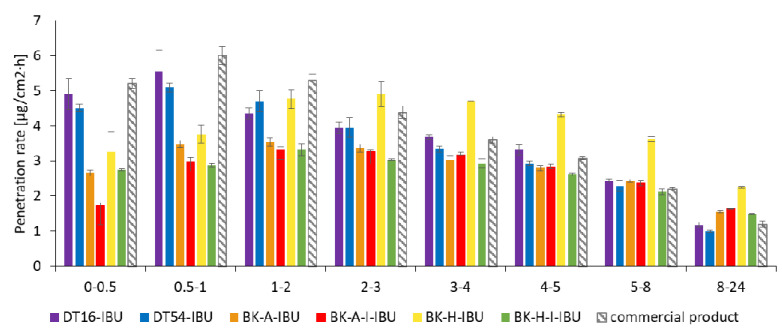
The penetration rate of IBU from acrylic PSAs during the 24 h penetration. Each point represents the mean ± SD (*n* = 3).

The penetration rate determined at each time interval is presented in Figure 5. Generally, the highest penetration rate to the acceptor fluid was observed in the first hours of the study for all samples. The DT-16, DT-54, and commercial products exhibited a burst effect, releasing the highest dose within 1 h. These patches in time 1–2 h released 19.38%, 18.87%, and 18.29% for the commercial product, DT16-IBU, and DT54-IBU, respectively. Then, a sharp drop was observed during the following hours of testing, while between 8-24 h it was only from 3.88% for a commercial product to 4.04% for DT16-IBU. In contrast, the IBU located in acrylic adhesives penetrated at a constant rate without a burst release in the first hours of the study. In this case, the permeation rate remained at a similar level from 2 to 8 h and was on average 14.24%. It was observed, after 8 h the penetration rate was usually twice as high as compared with commercial products and was 7.72% (BK-A-I-IBU), 7.14% (BK-H-IBU), 7.04% (BK-H-I-IBU), and 6.80% (BK-A-IBU)—Figure 5.

**Figure 6:**
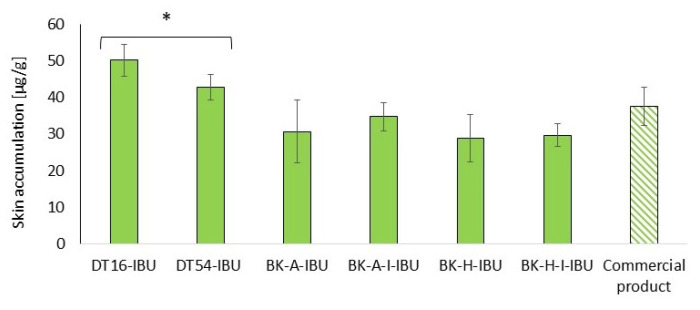
Accumulation in the skin of IBU during the 24 h penetration. Each point represents the mean ± SD (*n* = 3), α = 0.05. For * *p* < 0.0001 versus the control (commercial product). The statistically significant difference was estimated using the ANOVA test (Tukey’s test).

Our study also assessed the accumulation of IBU in the skin after 24-h penetration. Our study also assessed the accumulation of IBU in the skin after 2table4-h penetration. Figure 6 shows the mass of IBU that accumulated in pigskin in 24 h, expressed in μg IBU·g^−1^ of skin.

All of the patches used caused the accumulation of the drug in the skin. The highest statistically significant accumulation in the skin was for IBU released from DT16-IBU (50.08 ± 4.43) and DT54-IBU (42.74 ± 3.38), while, for our acrylic pressure-sensitive adhesives it was in the range from 28.82 ± 6.48 to 29.63 ± 3.14 μg IBU·g^−1^ of skin—Figure 6. 

## 3. Materials and Methods

### 3.1. Materials

The following components were used for the preparation of acrylic PSA: 2-ethylhexyl acrylate (2-EHA) (POLY-CHEM BmbH, Bitterfeld-Wolfen, Germany), ethyl acrylate (BASF, Ludwigshafen, Germany), isobornyl methacrylate (IBOMA, Evonik Industries AG, Essen, Germany), acrylic acid (AA), 2-hydroxyethyl acrylate (HEA), ethyl acetate, and AIBN (2,2′-azo-diisobutyonitrile) are purchased from BASF (Ludwigshafen, Germany) and 4-acryloyloxy benzophenone (ABP) (Chemitec, Scandiccy, Italy). The composition of acrylate polymers that are inherently pressure-sensitive is a combination of soft (low Tg), hard (high Tg), and monomers with functional groups. The monomers used in the research work were divided in terms of their function and presented in Table 5.

The commercial polyacrylate adhesives were also used in the research i.e., DURO-TAK 387-2516 (DT16; viscosity: 2.95 Pa·s; SWC: 43.5%) and DURO-TAK 378-2054 (DT54; viscosity: 1.46 Pa·s; SWC: 49.7%).

The following component was also used to prepare transdermal patches: ibuprofen (99%) (IBU) was obtained from Sigma Aldrich (Steinheim am Albuch, Germany). 

Other reagents used in the study, used for the permeation tests: PBS buffer pH 7.4 (Merck, Darmstadt, Germany). Orthophosphoric acid (98%) was high purity obtained from Chempur (Piekary Śląskie, Poland). Acetonitrile (≥99.9%) and methanol (99.9%) for HPLC gradient grade were provided by Sigma-Aldrich (Steinheim am Albuch, Germany). Anhydrous potassium dihydrogen phosphate (99%) (KH_2_PO_4_) was provided by Merck (Darmstadt, Germany).

### 3.2. Synthesis of Acrylic PSA

**Figure 7:**
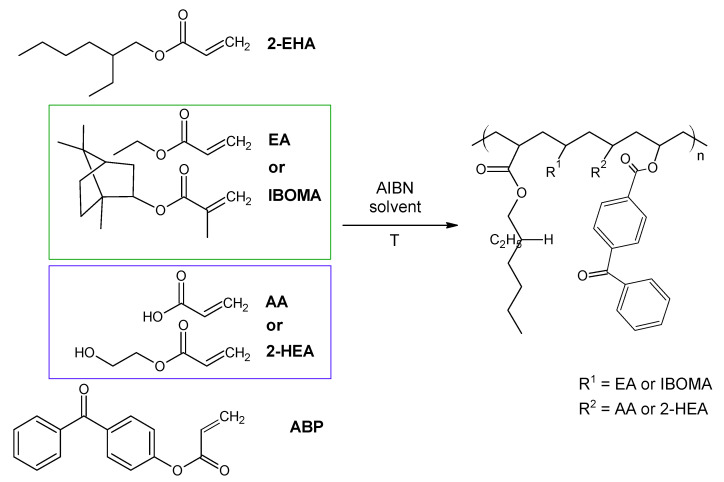
The schematic representation of the acrylic copolymers synthesis.

**Figure 8:**
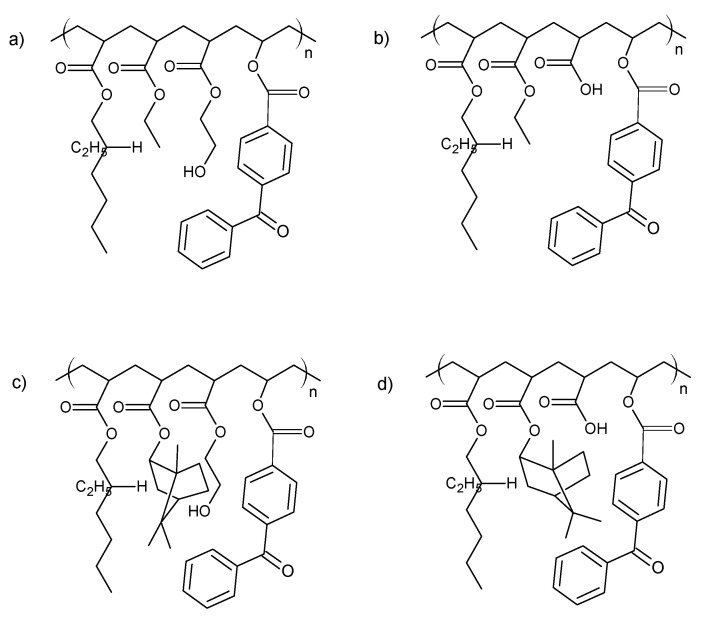
The theoretical structure of synthesized acrylic copolymers for transdermal patches ((**a**)—BK-A, (**b**)—BK-H; (**c**)—BK-A-I; (**d**)—BK-H-I).

The investigated acrylic PSA was synthesized in ethyl acetate (50 wt.% polymer content) from 80 wt.% 2-ethylhexyl acrylates, 15 wt.% of monomer increasing T_g_ (hard), 5 wt.% of monomers containing functional groups, and 0.5 wt.% of unsaturated copolymerizable acryloyloxyphotoinitiator (Figure 7, Table 6). The polymerization process was carried out under the following conditions: 2 h dosage time of monomers mixture and 5 h post-reaction time at presence of 0.1 wt.% radical starter azobisisobutyronitrile (AIBN) at the temperature of 78 °C (boiling point of ethyl acetate). The characterization of reaction mixtures and basic properties of acrylic copolymers is presented in Table 6, in turn, the theoretical structures of synthesized acrylic copolymers are shown in Figure 8. Supplementary Figures S13–S16 in the Supplementary Materials present the DSC curves of obtained acrylic copolymers.

**Figure 9:**
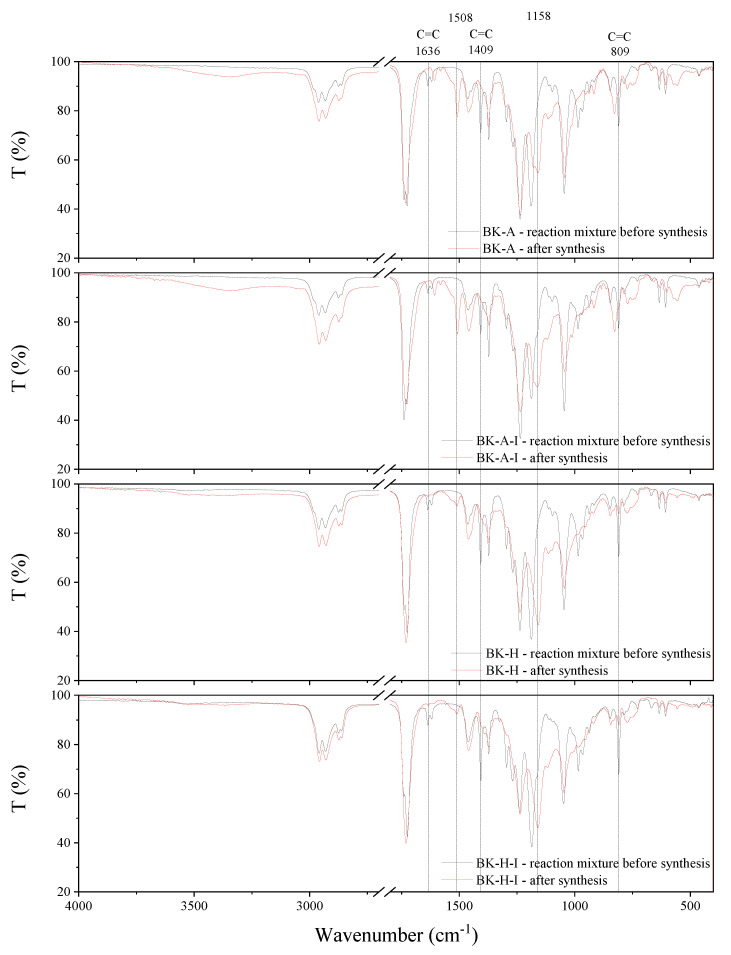
FT-IR spectra of synthesized acrylic copolymers before and after synthesis.

The synthesis behavior of the PSAs was studied by FT-IR spectroscopy (Figure 9). Acrylate groups take part in the reaction, hence the complete disappearance of sharp peaks at 1636 cm^−1^, 1409 cm^−1^, and 809 cm^−1^, which can be attributed to the strong absorbance of unsaturated bonds between carbon atoms (C=C), is observed after the synthesis. This confirms that the copolymerization reaction has taken place and obtaining copolymers with complete conversion of acrylate groups. Moreover, after the synthesis, new peaks at 1508 cm^−1^ and 1158 cm^−1^ are observed in the spectrum, which is probably responsible for the formation of ester bonds.

### 3.3. Preparation of Adhesive Films

Both commercial and synthesized acrylate copolymers constituted the adhesive matrix of the transdermal patches. First, adhesive films without drugs were prepared. For this purpose adhesives were coated (250 µm) on a polyester film. In the next stage, the obtained polymer layers from commercial adhesives were thermally cross-linked for 10 min at 110 °C. Whereas the obtained adhesives crosslinked after drying 10 min at 110 °C with transfer mercury UV lamp (Aktiprint-mini-18-2; UV dose: 650 mJ/cm^2^) at various crosslinked times. Table 7 summarizes the cross-linking conditions and the characteristics of the adhesives obtained. The resulting adhesive film layer was covered with siliconized release paper.

Next, adhesive compositions were prepared based on the obtained acrylate copolymers and ibuprofen. The weight ratio of adhesive matrix to active substance was calculated based on the adhesive characteristics, i.e., solids content, the basis weight depends on the applied thickness of the adhesive film and the characteristics of the active substance, i.e., the molar mass and the initial assumption regarding the content of active substances in commercial products, i.e., 200 mg of active substance for the surface of the adhesive film equal to 140 cm^2^. The adhesive compositions in this series were prepared by dissolving the active substance in ethyl acetate and then adding the mixture to the adhesive matrix. The obtained adhesive compositions were coated (250 µm) on a polyester film and crosslinked after 10 min at 110 °C with a transfer UV lamp (Aktiprint drying-mini-18-2; UV dose: 12 × 650 mJ/cm^2^). Table 8 summarizes the cross-linking conditions and the characteristics of the adhesive film obtained. The resulting adhesive film layer was covered with siliconized release paper.

### 3.4. Characterization and Performance Evaluation of Acrylic PSAs

The viscosity of the obtained acrylate copolymers was determined with a Bohlin Visco 88 (Malvern Panalytical) viscometer. The measurement was carried out at a temperature of 20 °C using the C14 geometry at a speed of 20 rpm.

Solid weight content determined in accordance with ISO 3251 (140 °C, 30 min) using a moisture analyzer (Radwag MAX 60/NP).

The coat weight of the cross-linked adhesive films (after evaporation of the solvent and after UV irradiation) was measured with a circular punch 1009 with an area of 10 cm^2^ (Karl Schröder KG, Weinheim, Germany).

DSC analyzing technique with differential calorimeter Q-100 (TA Instruments, New Castle, DE, USA, 2004) was employed to determine the glass transition temperature of adhesive. Samples were subjected to a heating cycle from −80 °C to +100 °C with a heating rate of 10 °/min.

Thermal stability was determined by means of thermogravimetric analyses conducted using thermomicrobalance TG 209 F1 Libra by Netzsch. Samples of approximately 5 mg weight were heated at a rate of 10 °/min in the oxidative atmosphere (nitrogen (protective gas): 10 cm^3^/min, air: 25 cm^3^/min) and a temperature range of 25 to 1000 °C. Onset decomposition temperature was determined from the intersection of TG curve tangents. The temperatures corresponding to the fastest sample weight loss were determined from the first derivative of the TG curve (DTG curve).

### 3.5. Evaluation of Self-Adhesive Properties

For the coated, cross-linked adhesive, the following self-adhesive properties were tested: tack, adhesion, and cohesion at different temperatures. For this purpose, international standards AFERA and FINAT were used. The shear strength was tested in accordance with FINAT FTM 8, adhesion according to AFERA 4001, and tack according to AFERA 4015. Tests were carried out on a Zwick/Roell Z-25 testing machine. 

The shear strength is a measure of the cohesiveness or internal strength of an adhesive. It is based upon the value of force required to pull an adhesive strip from a standard flat surface in a direction parallel to the surface to which it has been affixed with a definite pressure [35]. The shear strength test of the obtained adhesive tapes was tested at room temperature. The 2.5 × 2.5 cm adhesive film was applied to the steel plate and after about 10 min the other end of the strip was loaded with a one-kilogram weight. According to the standard, cohesion is the time after which the adhesive film will separate from the steel plate, under a load of 1 kg [36].

The peel adhesion is a force required to remove a coated flexible pressure-sensitive adhesive sheet material from a test panel. The peel adhesion measurement was performed on the Zwick/Roell Z010 testing machine at room temperature. The adhesive films 2.5 × 12.7 cm were obtained after crosslinking adhesive onto a standard steel plate. After 20 min the plate was placed in the lower holder of the testing machine, while the second part was placed in the upper holder horizontally other lend to the steel plate. The test involved removing the adhesive tape at an angle of 180 ° with a jaw speed of 300 mm/min. During the measurement, the force that must be used to peel the adhesive film from the steel plate was measured [36].

The tack measurement was carried out according to the AFERA 4015 method. According to this method, the force necessary to separate (at 100 mm per minute) a plate with a defined area and loop covered with adhesive 1-inch (about 2.5 cm) wide and about 7-inch (about 17.5 cm) long is tested. The tack test was carried out using a Zwick Roell Z010 testing machine. A sample of pressure-sensitive adhesive was placed in the jaw of a testing machine, which, when falling, caused contact with the metal plate [36,37].

### 3.6. In Vitro Skin Permeation Studies

The permeation experiments were done by using Franz diffusion cells (Phoenix DB-6, ABL&E-JASCO, Wien, Austria) with diffusion areas of 1 cm^2^. The acceptor chamber was 8 cm^3^ and was filled with PBS solution (pH 7.4). In each diffusion unit, a constant temperature of 32.0 ± 0.5 °C [38]. The acceptor chamber content was stirred with a magnetic stirring bar at the same speed for all cells. In the experiment, abdominal porcine skin coming from the local slaughterhouse was used. The porcine skin has similar permeability to human skin. Numerous histopathological studies confirmed its similarity to human skin [39,40].

The fresh abdominal porcine skin was washed in PBS buffer pH 7.4 several times. The skin of 0.5 mm in thickness was dermatomed. The skin was then divided into 2 cm × 2 cm pieces. The skin samples were wrapped in aluminum foil and stored in a freezer at −20 °C until use, not longer than three months. This frozen storage time was safe to keep skin barrier properties [41]. On the day of the experiment, the skin samples were slowly thawed at room temperature for 30 min and were hydrated by PBS pH 7.4 [42,43,44].

The skin was mounted on the donor chamber. The undamaged skin pieces with an even thickness were chosen for experiments. The integrity of the skin has been examined by checking its impedance (see Section 3.7).

The patches were cut to a 1 cm^2^ size that fitted the surface area of the donor compartment and covered the entire epidermal interface. Each patch was weighed an analytical balance and next glued to the skin attached to the donor chamber.

The experiment was carried for 24 h. The samples were reported after 0.5 h, 1 h, 2 h, 3 h, 4 h, 5 h, 8 h and 24 h of stirring. After this time aliquots of the acceptor fluid (0.3 cm^3^) were withdrawn and refilled with fresh buffer at the same pH. The IBU concentrations in the acceptor phase were measured by HPLC. The cumulative mass (µg/cm^2^) was calculated based on this concentration. The flux (in µg/cm^2^·h) of the ibuprofen and its derivatives through the pigskin into acceptor fluid was determined as the slope of the plot of cumulative mass in the acceptor fluid versus time.

Based on the obtained results of the in vitro efficiency permeation of ibuprofen, skin permeation parameters such as flux (J_SS_), apparent permeability coefficient (K_P_), lag time (L_T_), diffusion coefficient in the skin (D), skin partition coefficient (K_m_), and percent drug permeated after 24 h (Q_%24 h_) were determined. The steady-state fluxes (J_SS_) of ibuprofen and its derivatives through the skin were calculated from the slope of the plot of cumulative mass in the acceptor phase over time and were expressed as the amount of active ibuprofen per skin area and time (μg IBU/cm^2^·h). Lag time (L_T_) was determined by extrapolating the equation.

### 3.7. Skin Integrity

Skin impedance was measured using an LCRmeter4080 (Conradelectronic, Hirschau, Germany), which was operated in parallel mode at an alternating frequency of 120 Hz (error at kΩ values < 0.5%). For the measure of skin impedance, a donor chamber with a capacity of 2 cm^3^ was installed. The tips of measuring probes were immersed in the donor and acceptor chamber, filled with PBS (pH 7.4) as described previously [45,46]. The only skin samples of impedance >3 kΩ were applied. These values are similar to the electrical resistance for human skin [46]. After impedance measurement, the donor chamber was removed [1,34,42].

### 3.8. Accumulation in the Skin

The accumulation of the tested substance in the skin after penetration was determined using a modification of the methods described by Ossowicz-Rupniewska et al., Janus et al., Haq et al. [1,34,42]. After 24 h the patches and skin samples were removed from the Franz diffusion cell. The skin samples were carefully rinsed in PBS solution at 7.4 pH and dried at room temperature. The skin samples were weighed, cut by the diffusion area (1 cm^2^), and minced using scissors. Next, skin samples were placed in 2 cm^3^ methanol and were incubated for 24 h at 4 °C. After this time skin samples were homogenized for 3 min using a homogenizer (IKA^®^T18 digital ULTRA TURRAX (Staufen im Breisgau, Germany)). The homogenate was centrifuged at 3500 rpm for 5 min. The supernatant was collected and analyzed using HPLC. Accumulation of the IBU in the skin was calculated by dividing the amount of the drug remaining in the skin by a mass of skin sample and was expressed in mass of ibuprofen per mass of the skin (μg/g).

### 3.9. Quantitative Determinations in Permeability Studies by HPLC

Liquid chromatography system (Knauer, Germany) in skin permeation experiments for determination of IBU concentration in acceptor fluid and accumulation in the skin consisted of the following units: Smartline model 1050 pump, model 2600 UV detector, Smartline model 3950 autosampler model, model ClarityChrom 2009 integrator. The chromatographic column was of 5 μm pore size, 125 × 4 mm column Hyperisil ODS (C9). The detection wavelength was 220 nm. The mobile phase of 0.02 mol/dm^3^ potassium dihydrogen phosphate-acetonitrile-methanol (53/40/7, *v*/*v*/*v*) was adjusted to pH 2.5 with orthophosphoric acid and with a flow rate of 1 cm^3^/min. The column temperature was set at 25 °C, and the injection volume was 20 mm^3^.

### 3.10. Statistical Analysis

Results are presented as the mean ± standard deviation (SD). A one-way analysis of variance was used (ANOVA). The significance of differences between individual groups was evaluated with Tukey’s test (α < 0.05). A cluster analysis was carried out to characterize the ibuprofen penetration from individual patches. On this basis, groups by similar penetration were selected. Statistical calculations were done using Statistica 13 PL software (StatSoft, Polska).

## 4. Conclusions

Several solutions and suspension-type patch formulations were developed for the transdermal delivery of ibuprofen. Based on our results, in particular, has been developed a transdermal drug delivery system as an adhesive patch based on the photoreactive acrylate copolymers was successful with commercially available PSAs and obtained new acrylic-based PSAs with biocomponent. A study found that ibuprofen can easily be released from fabricated patches and penetrate through pig skin membranes. In addition, a comparatively steady release of the IBU was observed, which may be of benefit in the design of transdermal patches containing drugs requiring sustained release kinetics. The criteria, such as the type of raw material, the available technologies, and the application, establish the basis for trends in the development of adhesives for medical products.

## Figures and Tables

**Table 1:** Self-adhesive properties of acrylic PSAs.

Sample Code	SWC (%)	Shear Strength	Adhesion [N/25 mm]	Tack [N]
DT16	99	>72 h	9.23	13.60
DT54	98	>72 h	13.60	14.00
BK-A (3xUV)	97	>72 h	9.84	13.60
BK-A (6xUV)	98	>72 h	7.09	13.60
BK-A (9xUV)	98	>72 h	7.07	13.10
BK-A (12xUV)	98	>72 h	6.98	11.20
BK-A (15xUV)	98	>72 h	6.47	6.47
BK-A-I (3xUV)	97	>72 h	13.00	14.60
BK-A-I (6xUV)	98	>72 h	10.60	13.80
BK-A-I (9xUV)	98	>72 h	9.97	13.10
BK-A-I (12xUV)	98	>72 h	9.67	12.90
BK-A-I (15xUV)	98	>72 h	9.93	11.20
BK-H (3xUV)	96	6 min/c.f.	4.44	8.34
BK-H (6xUV)	97	3 min/c.f.	2.63	7.55
BK-H (9xUV)	97	60 h/c.f.	2.29	6.77
BK-H (12xUV)	97	>72 h	1.83	6.60
BK-H (15xUV)	98	>72 h	1.83	5.44
BK-H-I (3xUV)	97	21 h/c.f.	14.00	16.30
BK-H-I (6xUV)	99	>72 h	10.50	12.12
BK-H-I (9xUV)	99	>72 h	7.45	12.03
BK-H-I (12xUV)	99	>72 h	7.32	11.80
BK-H-I (15xUV)	99	>72 h	7.13	11.30

SWC—Solid weight content determined via gravimetry; c.f.—cohesive failure.

**Table 2:** Self-adhesive properties of acrylic PSAs with ibuprofen (IBU).

Sample Code	SWC (%)	Shear Strength	Adhesion [N/25 mm]	Tack [N]
DT16-IBU	96	3 min/c.f.	15.7/c.f.	18.5
DT54-IBU	97	10 min/c.f.	11.9/c.f.	21.4
BK-A-IBU	91	4 min/c.f.	4.4/c.f.	6.5
BK-A-I-IBU	94	15 min/c.f.	19.5/c.f.	9.7
BK-H-IBU	93	1 h 3 min/c.f.	1.7/c.f.	5.1
BK-H-I-IBU	94	1 h 12 min/c.f.	9.3/c.f.	10.9

SWC—Solid weight content determined via gravimetry; c.f.—cohesive failure.

**Table 3:** Thermal stability of acrylic PSAs and PSAs with ibuprofen (IBU).

Sample Code	T_IDT_ [°C]	T_d_^50%^ [°C]	T_MDT_ [°C]
DT16	304.4	380.8	372.7
DT16-IBU	153.7	353.4	367.5
DT54	303.9	357.6	361.9
DT54-IBU	166.8	333.0	367.5
BK-A	326.6	371.1	376.7
BK-A-IBU	166.9	363.0	383.2
BK-A-I	222.9	346.6	373.5
BK-A-I-IBU	153.7	322.2	329.4
BK-H	219.7	340.5	341.7
BK-H-IBU	168.6	363.1	377.1
BK-H-I	299.4	352.8	388.7
BK-H-I-IBU	170.2	354.5	393.5

T_IDT_—onset decomposition temperature, T_d_^50%^—50% weight loss temperature. T_MDT_—maximum decomposition temperature.

**Table 4:** Skin permeation parameters for ibuprofen from different acrylic PSAs.

Sample Code	CM, µg/cm^2^	J_SS_, µg/cm^2^∙h	K_P_∙10^3^, cm/h	L_T_, min	D, cm^2^/h	K_m_∙10^3^	Q%_24 h_
DT16-IBU	28.440 ± 1.675	4.026	2.818	12.922	0.039	3.642	1.991
DT54-IBU	23.979 ± 0.547	4.702	3.291	1.231	0.406	4.053	1.679
BK-A-IBU	37.247 ± 0.585	3.794	2.656	7.324	0.068	1.945	2.615
BK-A-I-IBU	39.577 ± 0.251	3.816	2.671	15.109	0.033	4.036	2.770
BK-H-IBU	54.098 ± 0.596	5.338	3.737	14.079	0.036	5.261	3.787
BK-H-I-IBU	35.736 ± 0.275	3.541	2.478	8.481	0.059	2.102	2.502
Commercial product	28.817 ± 2.158	5.226	3.658	5.226	0.143	1.276	2.017

CM—the average cumulative mass of ibuprofen after 24 h permeation test; Jss—steady-state flux; KP—permeability coefficient; LT—Lag time; D—diffusion coefficient in the skin; Km—skin partition coefficient; Q%_24 h_—percent drug permeated after 24 h.

**Table 5:** Monomers used in the synthesis of acrylic PSA.

Monomer	Structure	Tg for Homopolymer [°C]	Function
2-ethylhexyl acrylate (2-EHA)	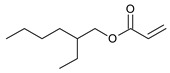	−70	Tackifying monomers (soft)
ethyl acrylate (EA)	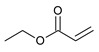	−24	Monomers increasing Tg (hard)
isobornyl methacrylate (IBOMA)	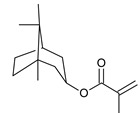	+105	Monomers increasing Tg (hard)
acrylic acid (AA)	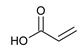	+106	Monomers containing functional groups
2-hydroxyethyl acrylate (HEA)	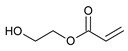	−15	Monomers containing functional groups
4-acryloyloxy benzophenone (ABP)	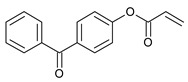		Unsaturated photoinitiator

T_g_—the glass transition temperature.

**Table 6:** The reaction mixtures and properties of the obtained acrylic copolymers.

PSA Sample	Monomer Formulation	Weight Ratio	SWC [%]	η [Pa·s]	T_g_ [°C]
BK-A	2EHA:EA:AA	80:15:5	61	2.95	−47.04
BK-A-I	2EHA:IBOMA:AA	80:15:5	70	5.05	−36.21
BK-H	2EHA:EA:HEA	80:15:5	65	2.99	−49.42
BK-H-I	2EHA:IBOMA:HEA	80:15:5	66	2.08	−41.48

SWC—Solid weight content determined via gravimetry; η—viscosity; T_g_—the glass transition temperature.

**Table 7:** The cross-linking conditions and the characteristics of acrylic PSAs.

Sample Code	Coat Weight [g/m^2^]	Thickness ^(1)^ [µm]	Temperature ^(2)^ [°C]/Time ^(3)^ [min]	UV Dose ^(4)^ [mJ/cm^2^]
DT16	24	250	110/10	
DT54	32	250	110/10	
BK-A (3 × UV)	38	250	110/10	3 × 650
BK-A (6 × UV)				6 × 650
BK-A (9 × UV)				9 × 650
BK-A (12 × UV)				12 × 650
BK-A (15 × UV)				15 × 650
BK-A-I (3 × UV)	54	250	110/10	3 × 650
BK-A-I (6 × UV)				6 × 650
BK-A-I (9 × UV)				9 × 650
BK-A-I (12 × UV)				12 × 650
BK-A-I (15 × UV)				15 × 650
BK-H (3 × UV)	46	250	110/10	3 × 650
BK-H (6 × UV)				6 × 650
BK-H (9 × UV)				9 × 650
BK-H (12 × UV)				12 × 650
BK-H (15 × UV)				15 × 650
BK-H-I (3 × UV)	40	250	110/10	3 × 650
BK-H-I (6 × UV)				6 × 650
BK-H-I (9 × UV)				9 × 650
BK-H-I (12 × UV)				12 × 650
BK-H-I (15 × UV)				15 × 650

(1) the thickness of the adhesive film; (2) temperature during drying of the adhesive film; (3) drying time of the adhesive film; (4) radiation dose during cross-linking of the adhesive film.

**Table 8:** The cross-linking conditions and the characteristics of acrylic PSAs with ibuprofen (IBU).

Sample Code	Coat Weight [g/m^2^]	Thickness ^(1)^ [µm]	Temperature ^(2)^ [°C]/time ^(3)^ [min]	UV Dose ^(4)^ [mJ/cm^2^]
DT16-IBU	30	250	110/10	
DT54-IBU	40			
BK-A-IBU	64	250	110/10	12 × 650
BK-A-I-IBU	53			
BK-H-IBU	77			
BK-H-I-IBU	52			

(1) the thickness of the adhesive film; (2) temperature during drying of the adhesive film; (3) drying time of the adhesive film; (4) radiation dose during cross-linking of the adhesive film.

## Data Availability

Most of the data are provided in this work and in Supplementary Data. Other data that support the findings of this study are available from the corresponding author upon reasonable request.

## Supplementary Materials

The following are available online at https://www.mdpi.com/article/10.3390/ijms222111840/s1, Figure S1: The TG and DTG curves of DT16, Figure S2: The TG and DTG curves of DT16-IBU, Figure S3: The TG and DTG curves of DT54, Figure S4: The TG and DTG curves of DT54-IBU, Figure S5: The TG and DTG curves of BK-A, Figure S6: The TG and DTG curves of BK-A-IBU, Figure S7: The TG and DTG curves of BK-A-I, Figure S8. The TG and DTG curves of BK-A-I-IBU, Figure S9: The TG and DTG curves of BK-H, Figure S10: The TG and DTG curves of BK-H-IBU, Figure S11: The TG and DTG curves of BK-H-I, Figure S12: The TG and DTG curves of BK-H-I-IBU, Figure S13: The DSC curve of BK-A, Figure S14: The DSC curve of BK-A-I, Figure S15: The DSC curve of BK-H, Figure S16: The DSC curve of BK-H-I.
