# Supplementary material for: Sustainable UV-Crosslinkable Acrylic Pressure-Sensitive Adhesives for Medical Application
Source: Int J Mol Sci. 2021 Oct 31;22(21):11840. doi: 10.3390/ijms222111840 (PMC8584108; doi:10.3390/ijms222111840)
Supplement: Supplementary file 1 [file ijms-22-11840-s001.zip › ijms-1421344-supplementary.pdf]

International Journal of *Molecular Sciences*

MDPI

Supplementary Materials

Sustainable UV-crosslinkable acrylic pressure-sensitive adhesives for medical application

Paula Ossowicz-Rupniewska [1,*], Paulina Bednarczyk [1], Małgorzata Nowak [1], Anna Nowak [2], Wiktoria Duchnik [2], Łukasz Kucharski [2], Joanna Rokicka [1], Adam Klimowicz [2], Zbigniew Czech[1]

[1] West Pomeranian University of Technology in Szczecin, Faculty of Chemical Technology and Engineering, Department of Chemical Organic Technology and Polymeric Materials, Piastów Ave. 42, PL-71065 Szczecin, Poland; bednarczyk.pb@gmail.com (P.B.); gosia.nowak.zut@gmail.com (M.N.); psa_czech@wp.pl (Z.C.)
[2] Pomeranian Medical University in Szczecin, Department of Cosmetic and Pharmaceutical Chemistry, Powstańców Wielkopolskich Ave. 72, PL 70111 Szczecin, Poland; anowak@pum.edu.pl (A.N.); wiktoria.duchnik@pum.edu.pl (W.D.); lukasz.kucharski@pum.edu.pl (Ł.K.); adklim@pum.edu.pl (A.K.)
* Correspondence: possowicz@zut.edu.pl; Tel.: +48 914494801

Citation: Ossowicz-Rupniewska, P.; Bednarczyk, P.; Nowak, M.; Nowak, A.; Duchnik, W.; Kucharski, Ł.; Rokicka, J.; Klimowicz, A.; Czech. Z. Sustainable UV-crosslinkable acrylic pressure-sensitive adhe-sives for medical application. *Int. J. Mol. Sci.* **2021**, *22*, 11840. https://doi.org/10.3390/ijms222111840

Publisher's Note: MDPI stays neutral with regard to jurisdictional claims in published maps and institutional affiliations.

Copyright: © 2021 by the authors. Submitted for possible open access publication under the terms and conditions of the Creative Commons Attribution (CC BY) license (https://creativecommons.org/licenses/by/4.0/).

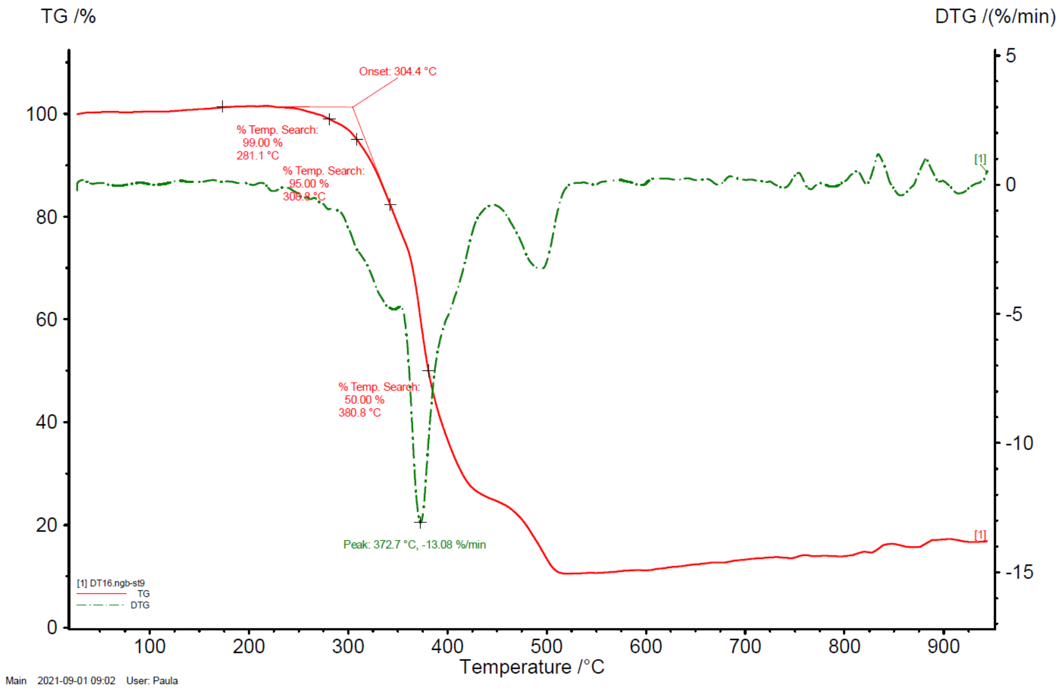

Figure S1. The TG and DTG curves of DT16.

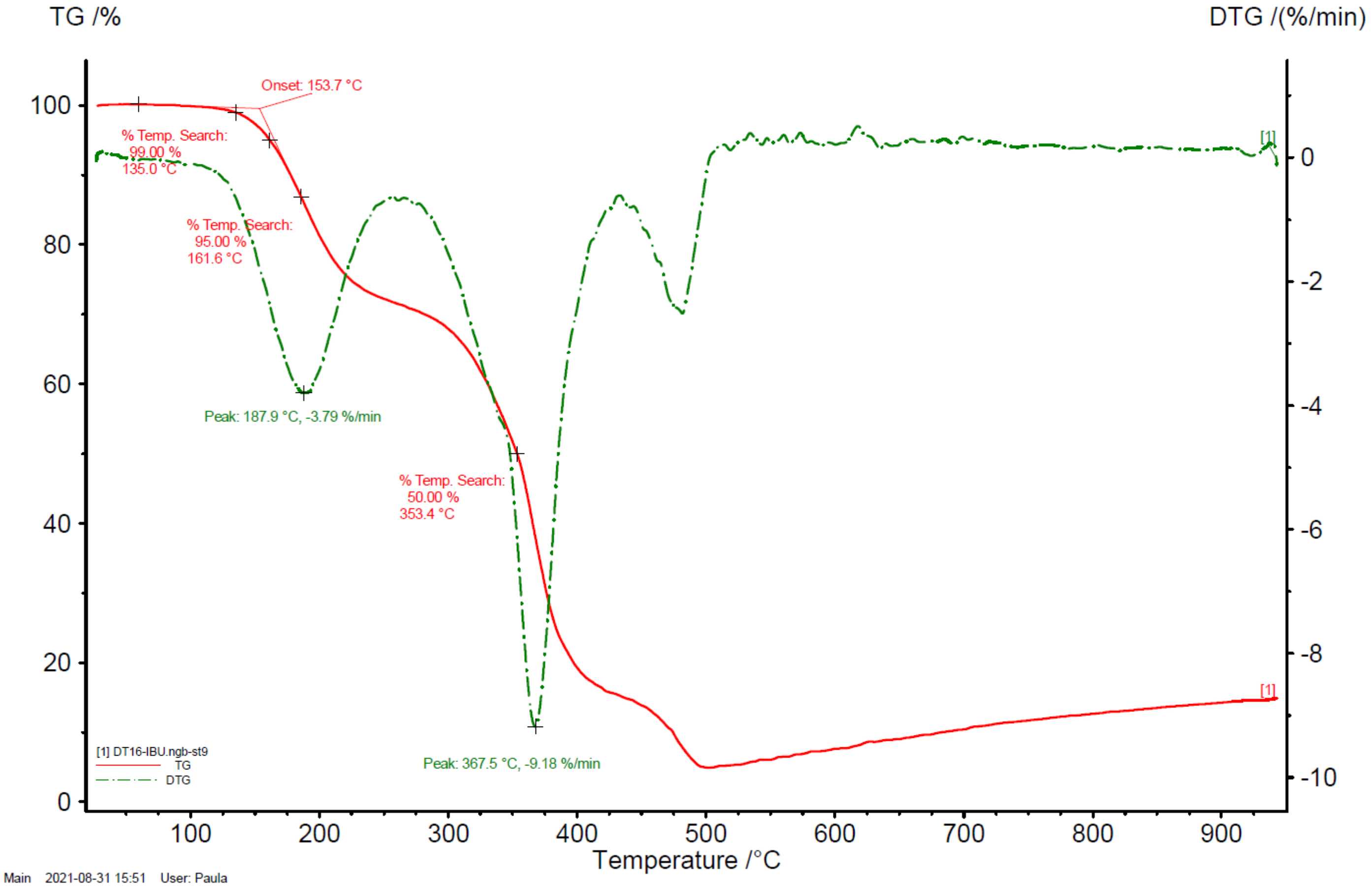

Figure S2. The TG and DTG curves of DT16-IBU.

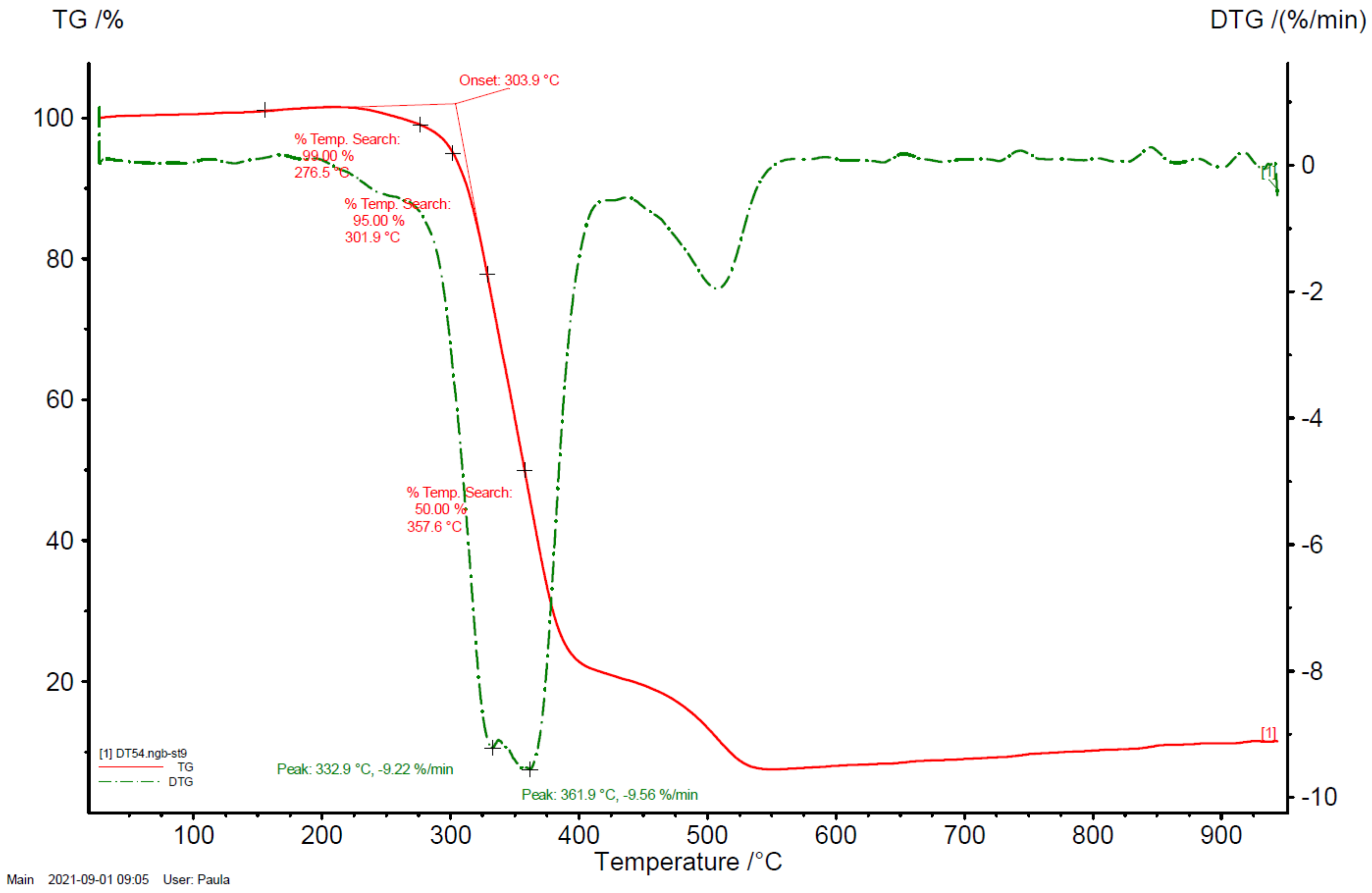

Figure S3. The TG and DTG curves of DT54.

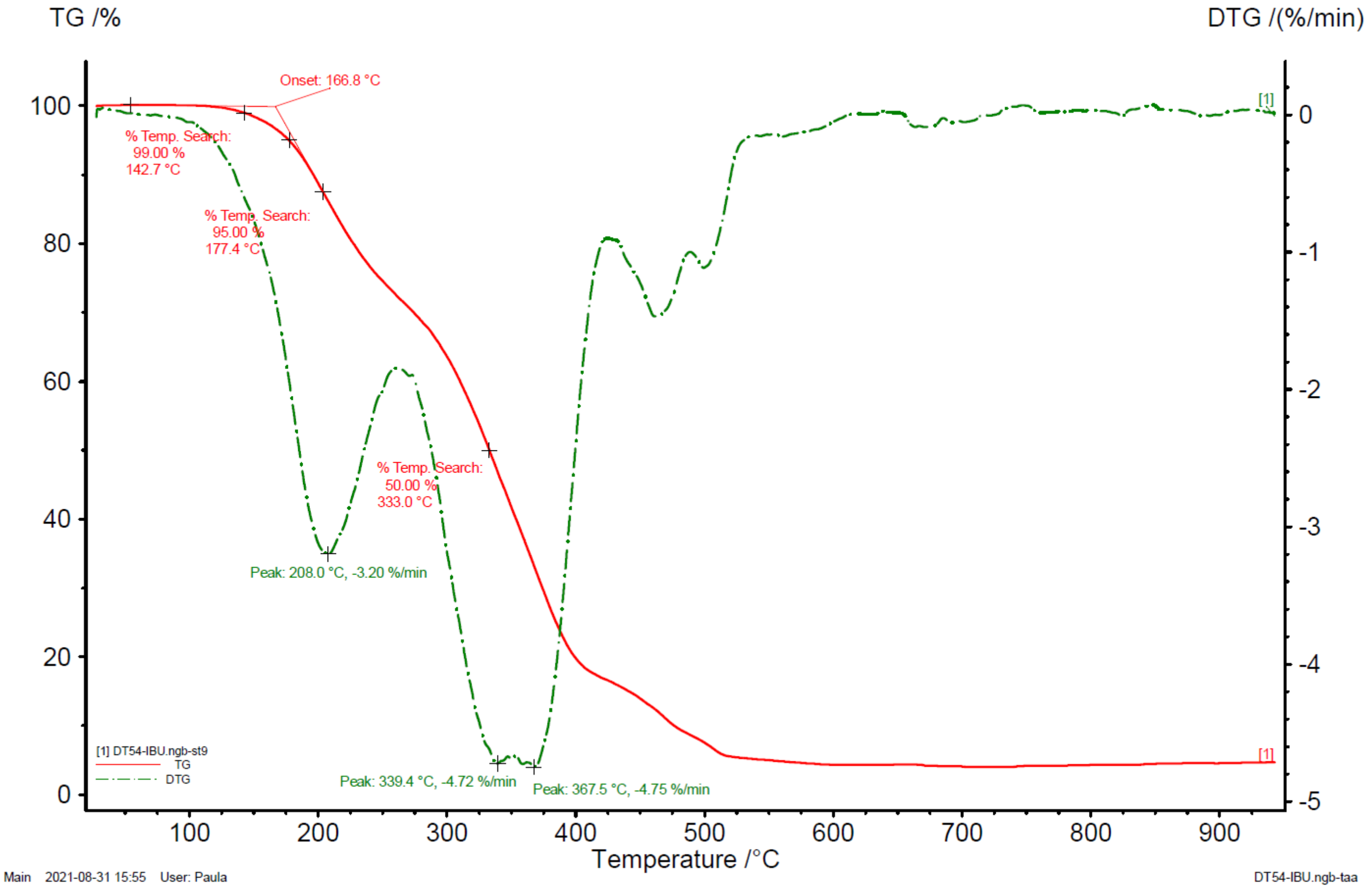

Figure S4. The TG and DTG curves of DT54-IBU.

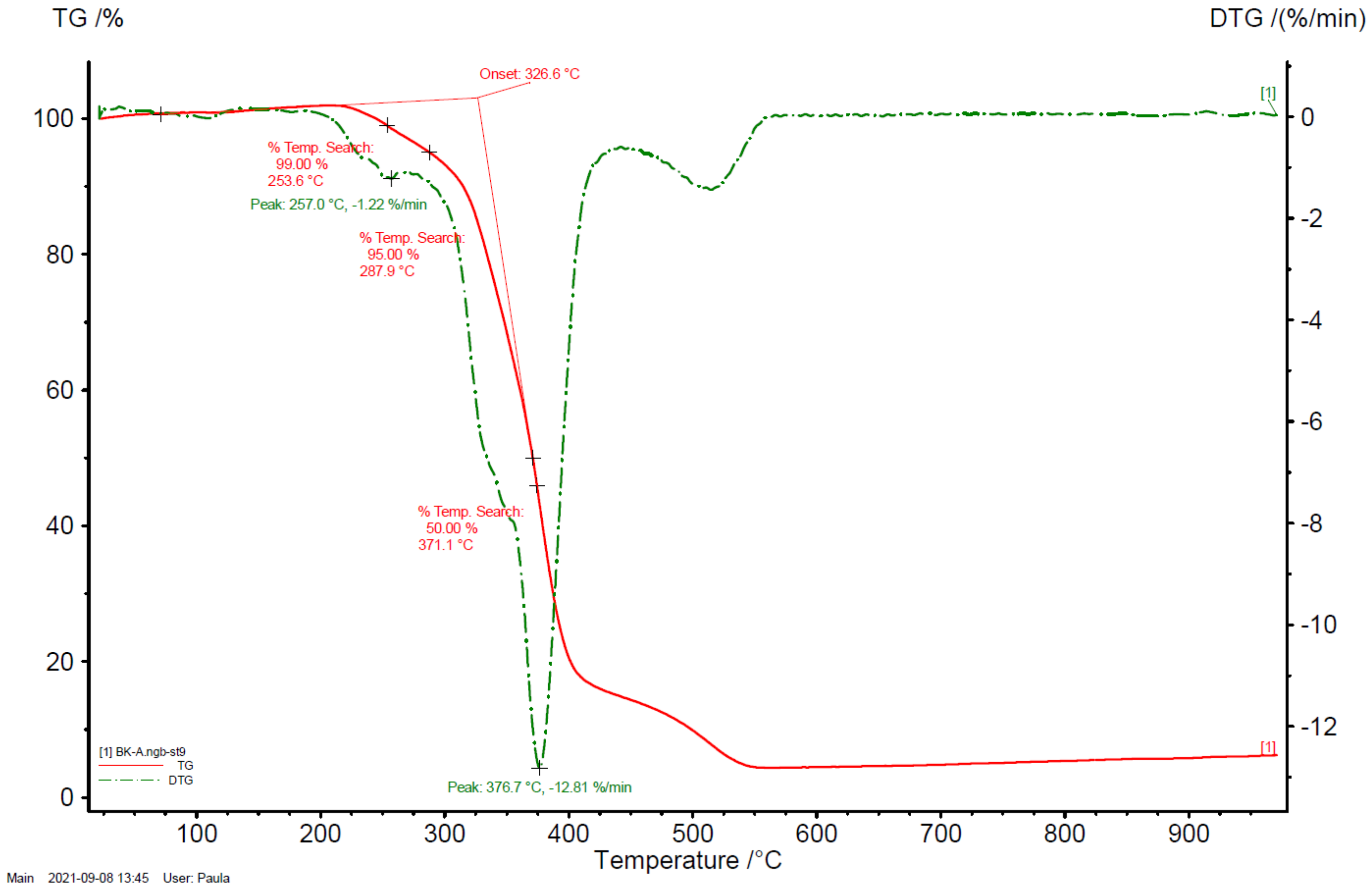

Figure S5. The TG and DTG curves of BK-A.

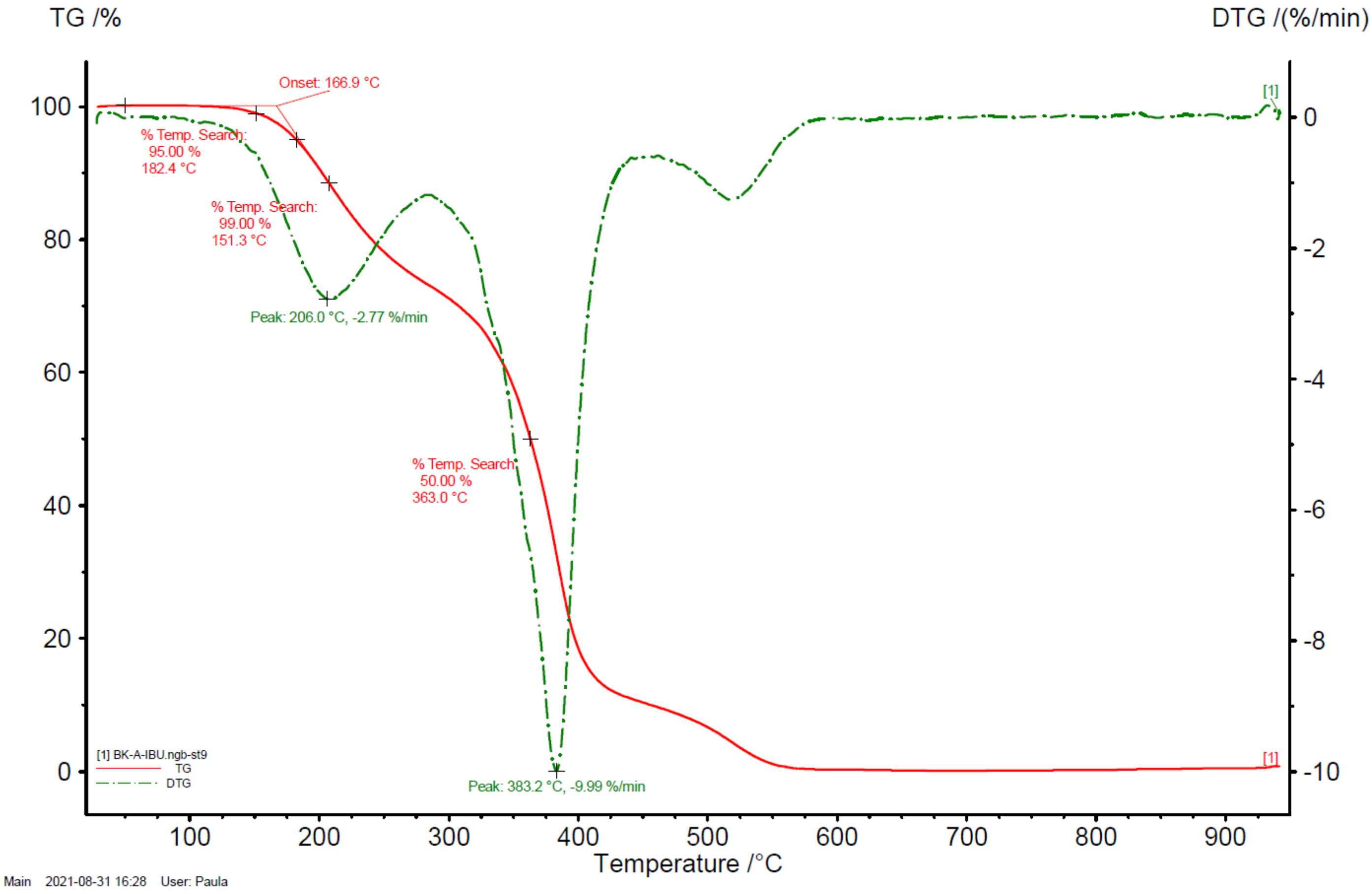

Figure S6. The TG and DTG curves of BK-A-IBU.

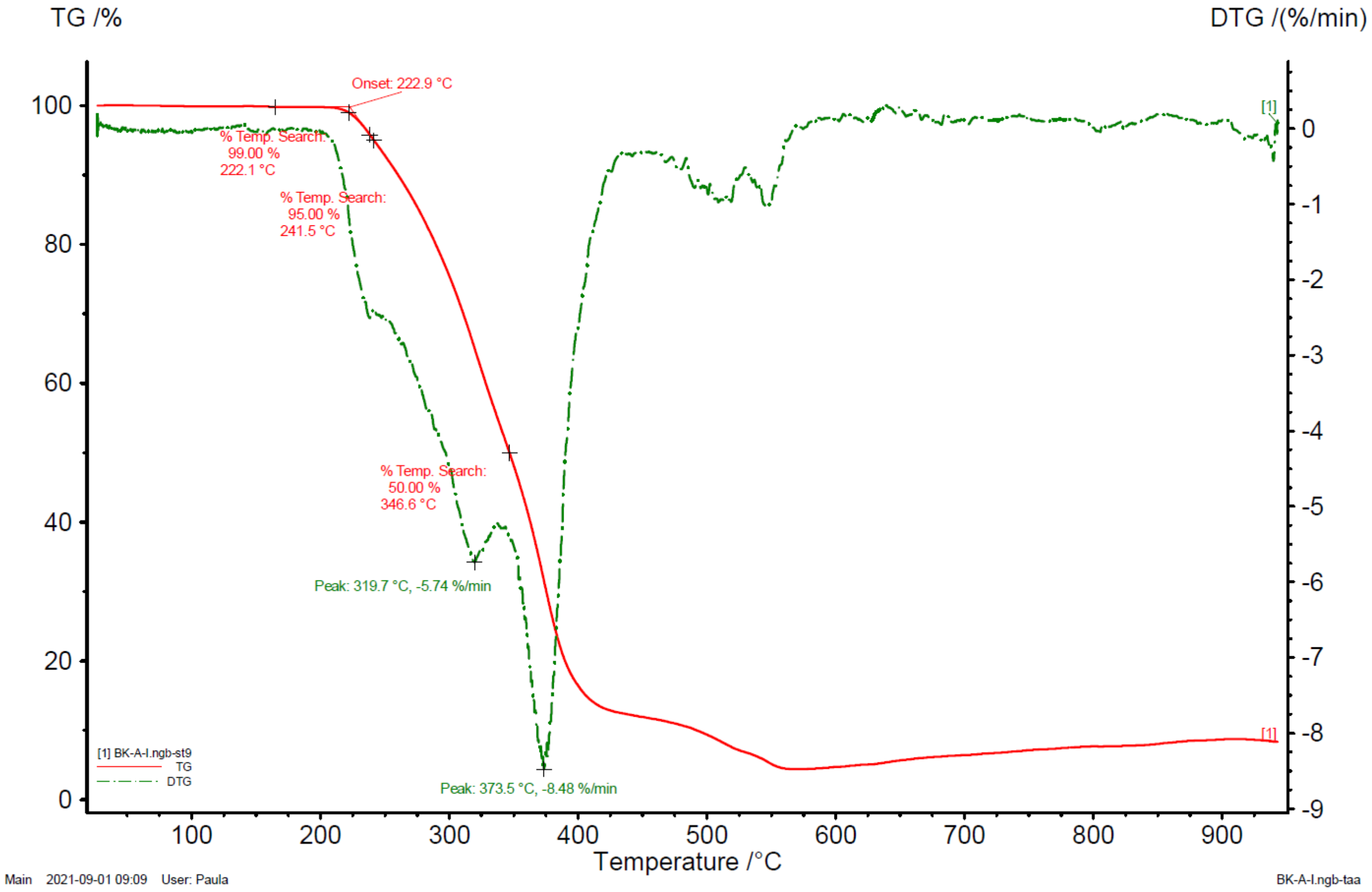

Figure S7. The TG and DTG curves of BK-A-I.

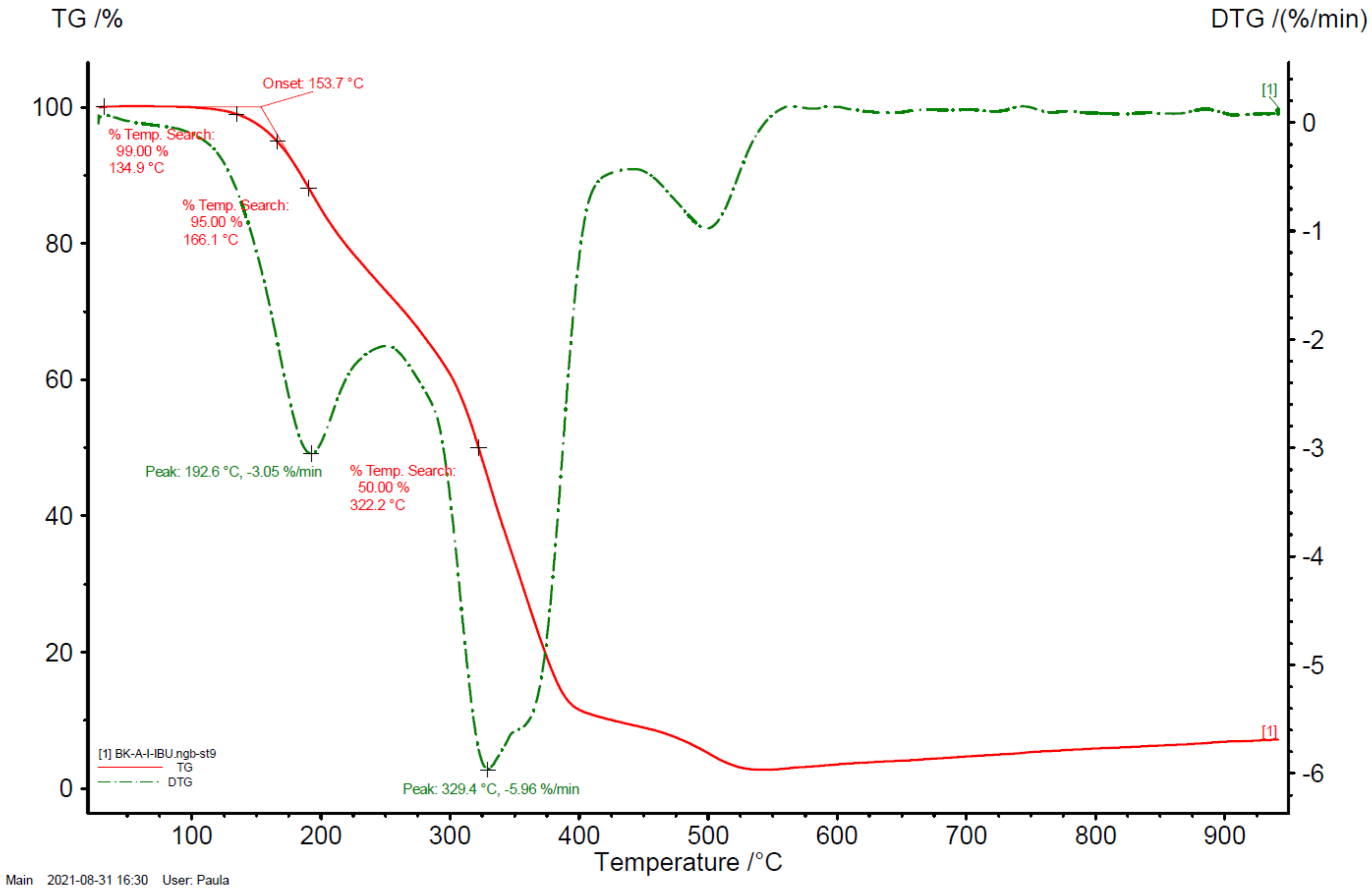

Figure S8. The TG and DTG curves of BK-A-I-IBU.

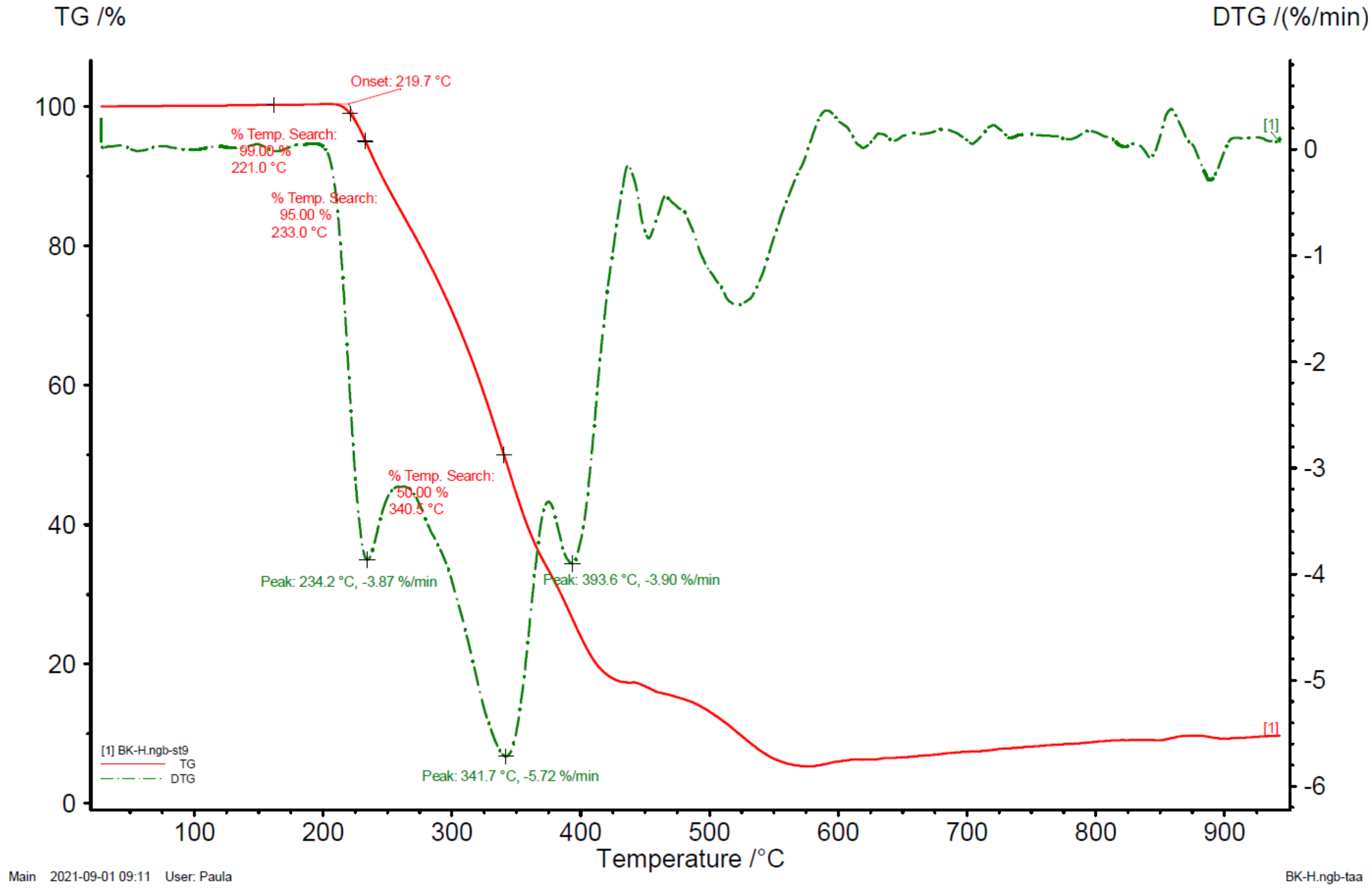

Figure S9. The TG and DTG curves of BK-H.

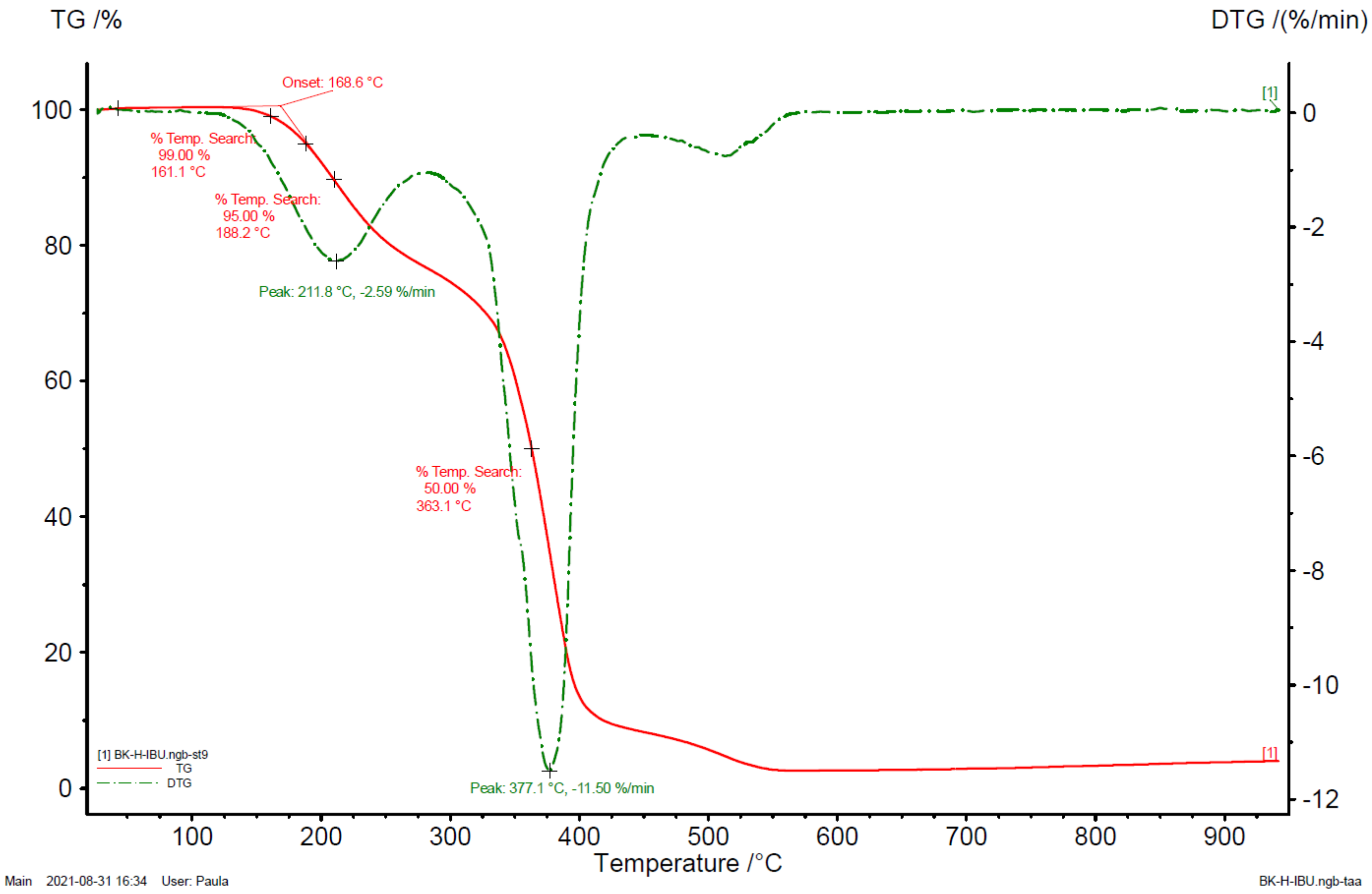

Figure S10. The TG and DTG curves of BK-H-IBU.

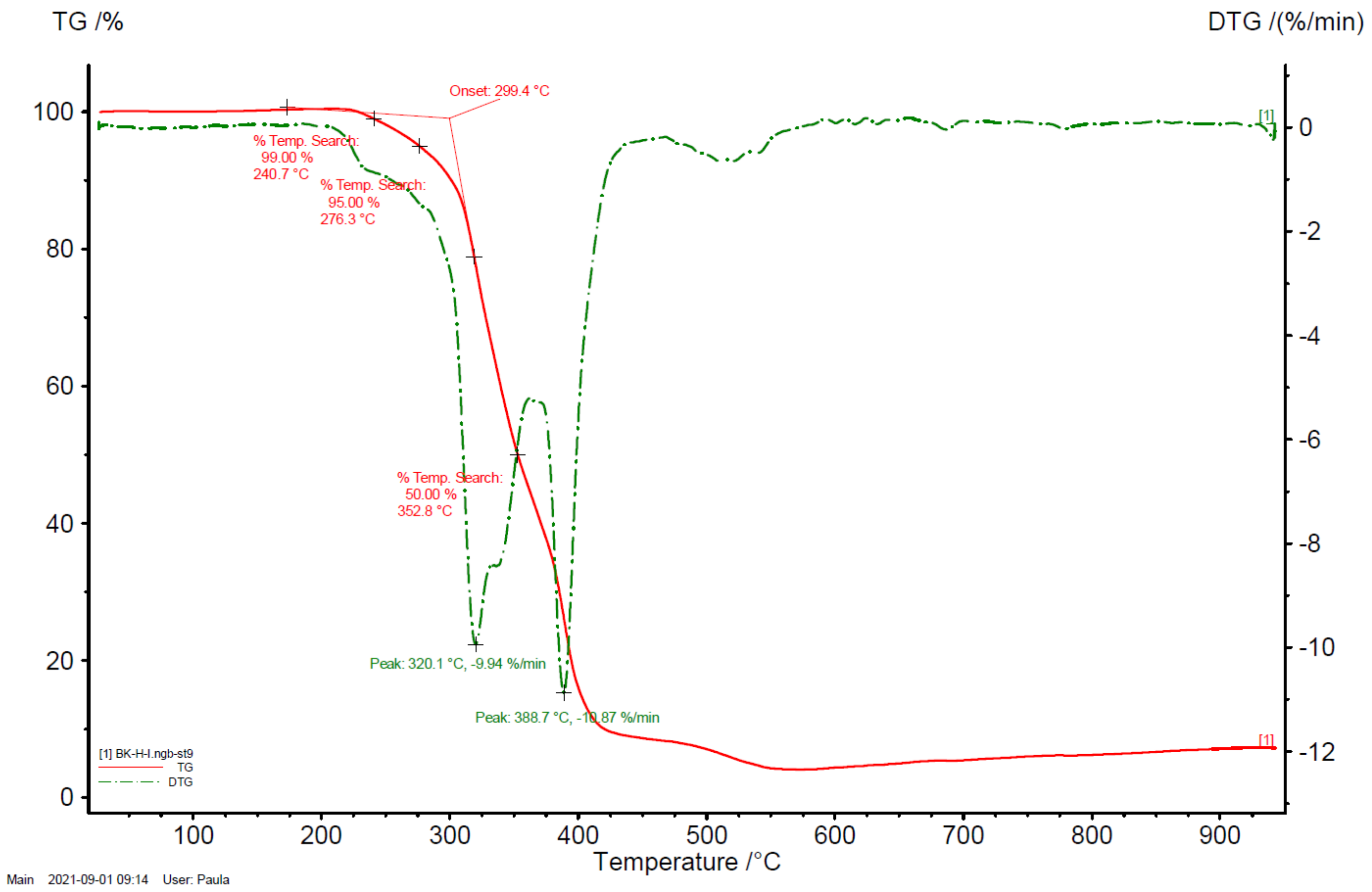

Figure S11. The TG and DTG curves of BK-H-I.

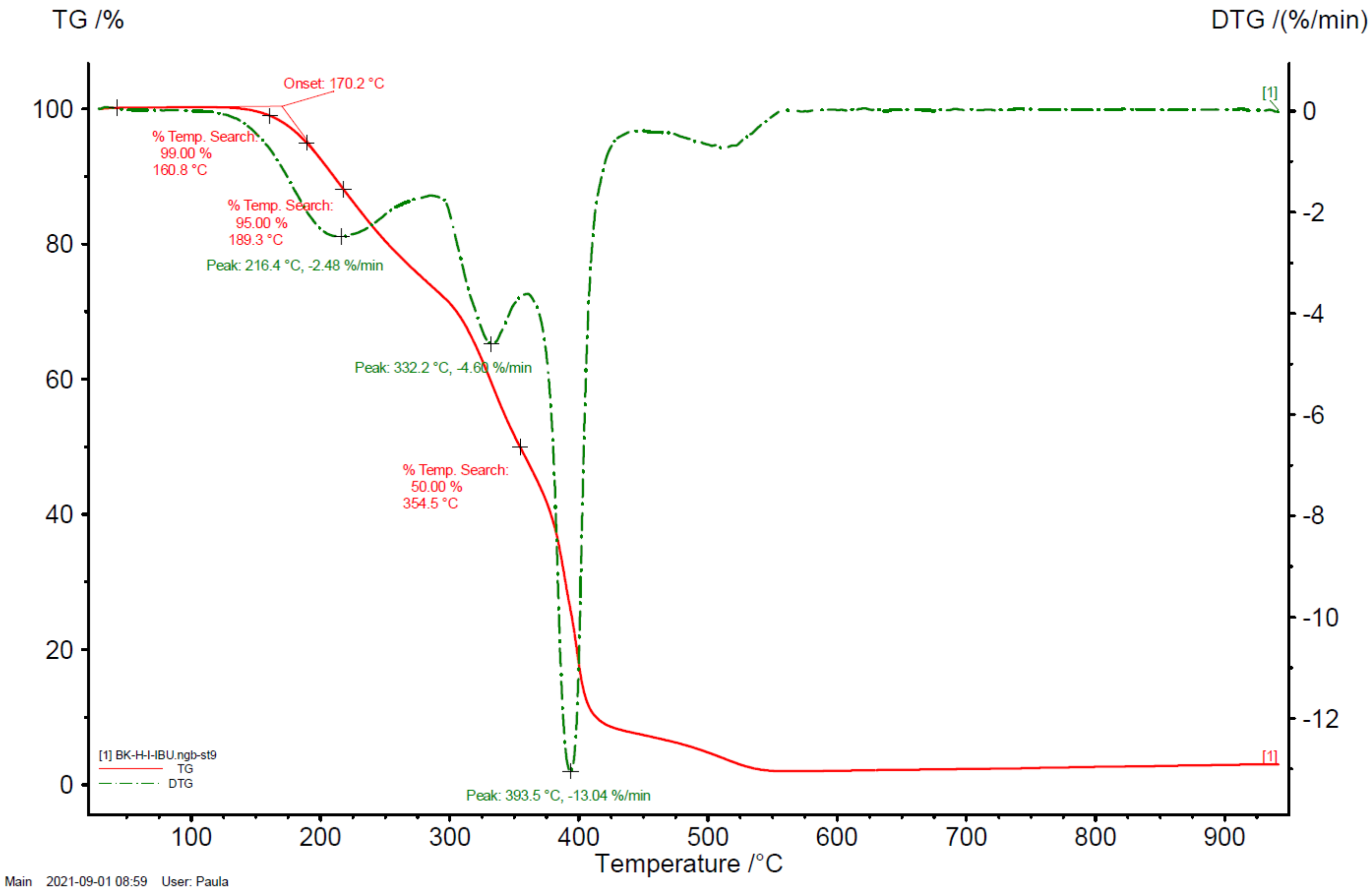

Figure S12. The TG and DTG curves of BK-H-I-IBU.

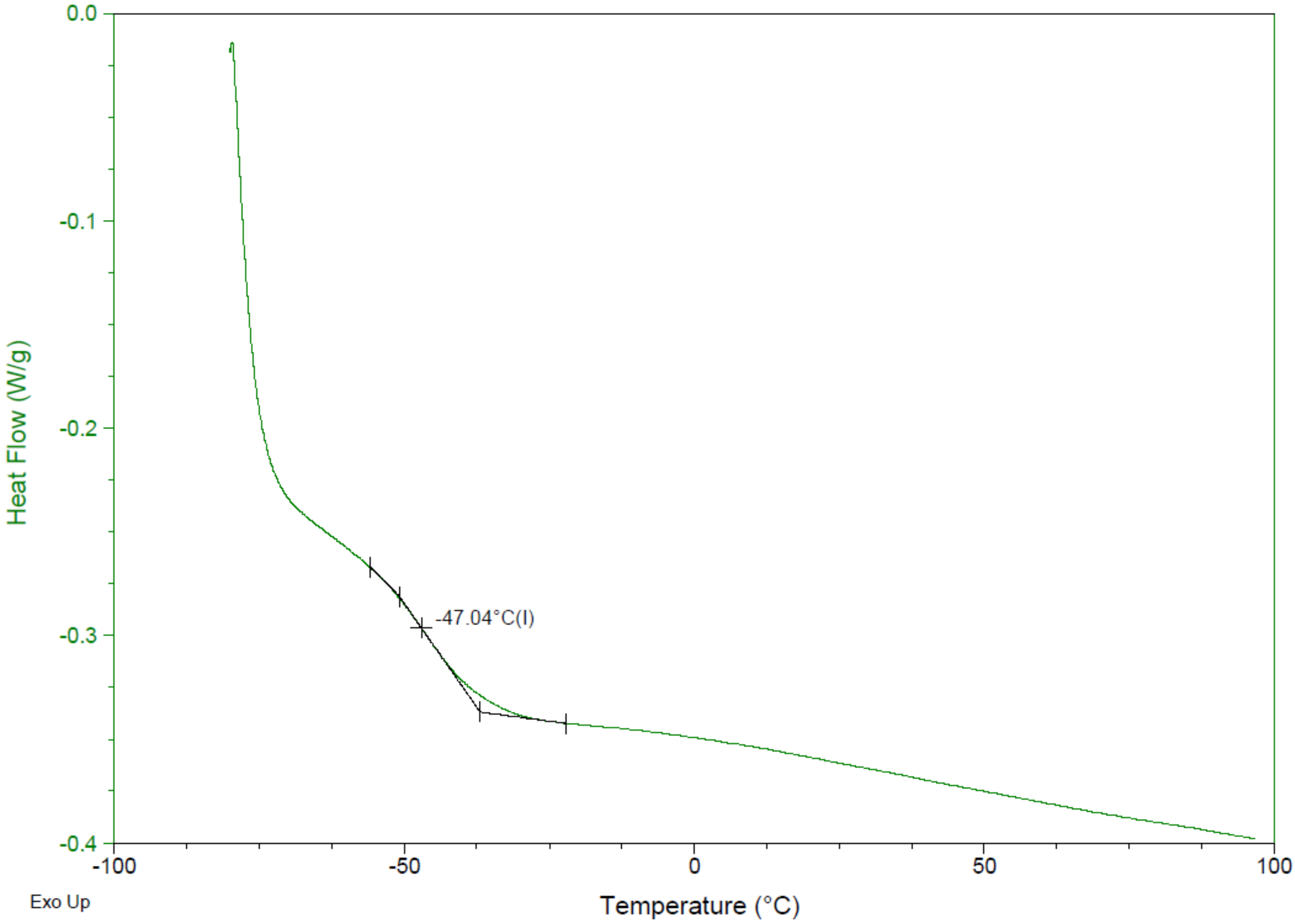

Figure S13. The DSC curve of BK-A.

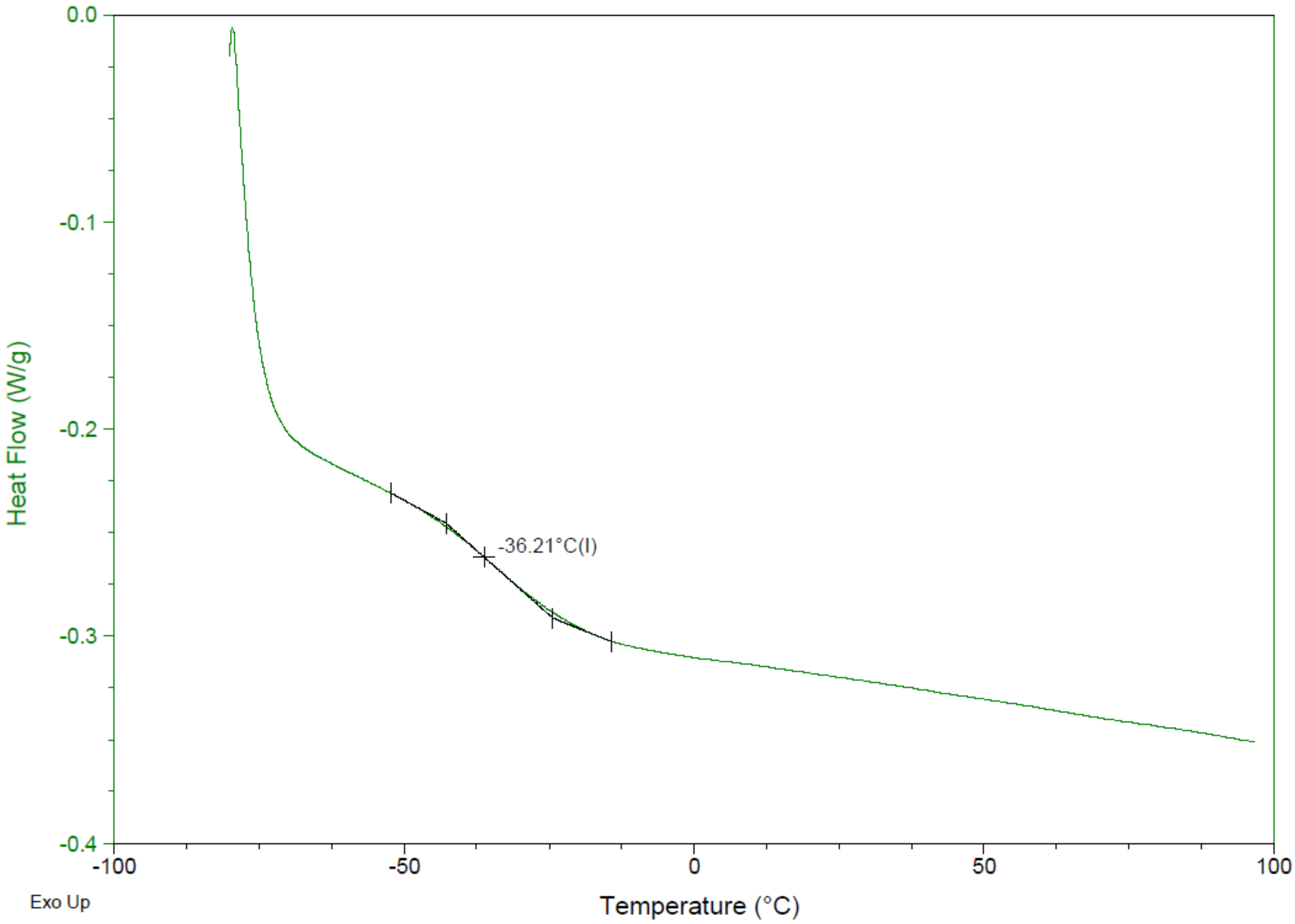

Figure S14. The DSC curve of BK-A-I.

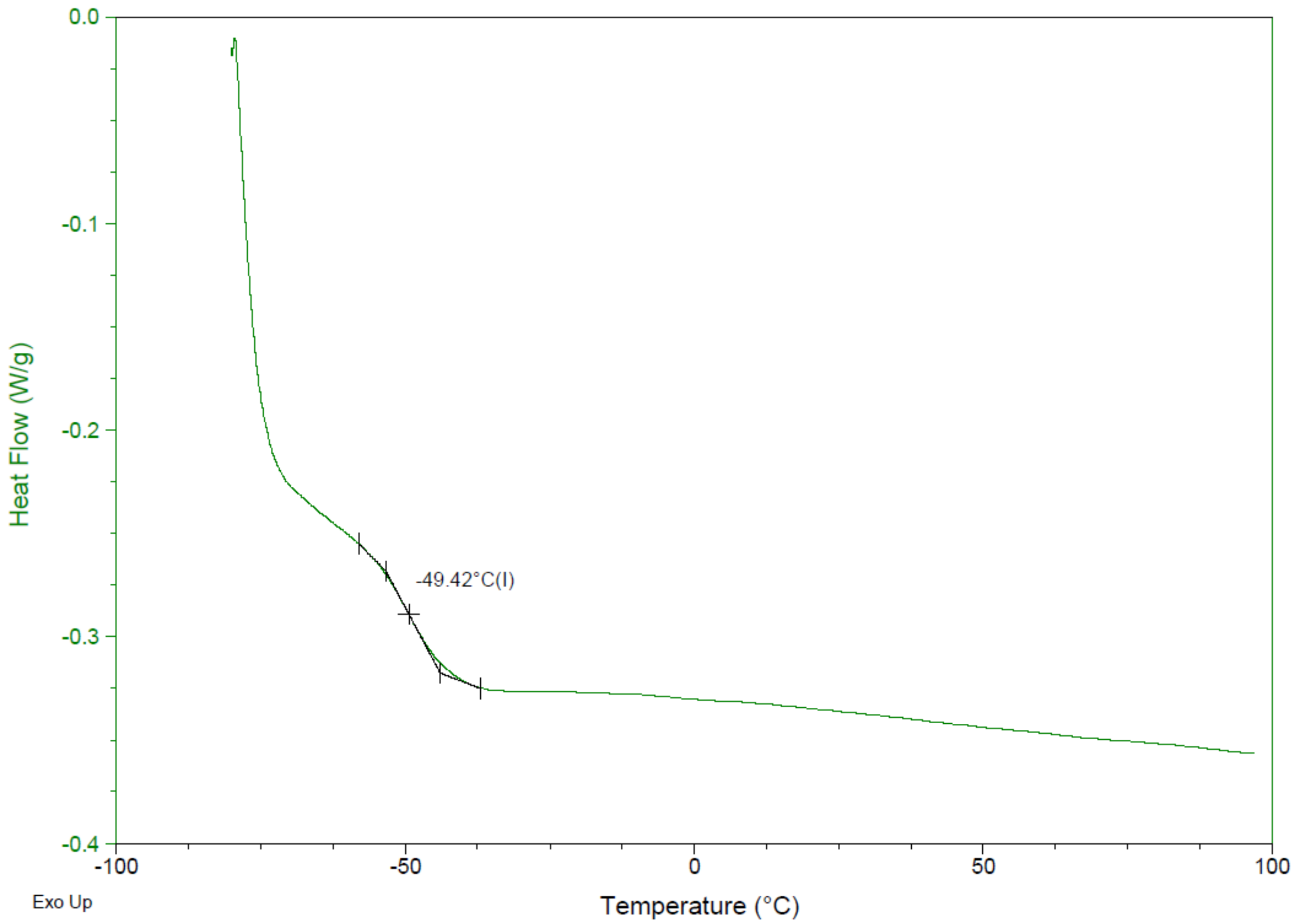

Figure S15. The DSC curve of BK-H.

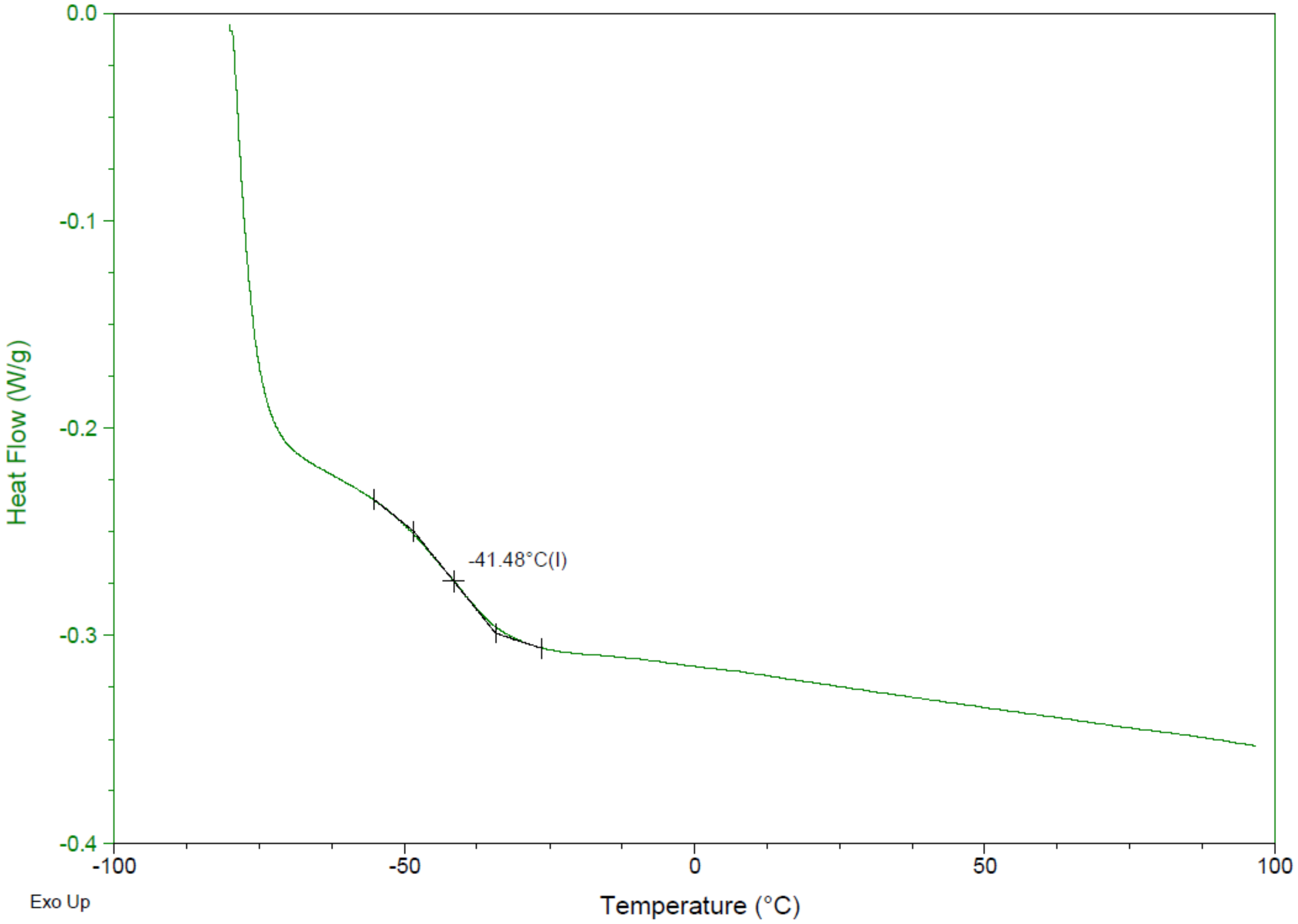

Figure S16. The DSC curve of BK-H-I.
